# Using Resistin, glucose, age and BMI to predict the presence of breast cancer

**DOI:** 10.1186/s12885-017-3877-1

**Published:** 2018-01-04

**Authors:** Miguel Patrício, José Pereira, Joana Crisóstomo, Paulo Matafome, Manuel Gomes, Raquel Seiça, Francisco Caramelo

**Affiliations:** 1 Laboratory of Biostatistics and Medical Informatics and IBILI - Faculty of Medicine, University of Coimbra, Azinhaga Santa Comba, Celas, 3000-548 Coimbra, Portugal; 2 Faculty of Medicine, University of Coimbra, Coimbra, Portugal; 3 Laboratory of Physiology, IBILI - Faculty of Medicine of University of Coimbra, Coimbra, Portugal; 4 Department of Complementary Sciences, Coimbra Health School - Instituto Politécnico de Coimbra, Coimbra, Portugal; 5 Department of Internal Medicine, University Hospital Centre of Coimbra, Coimbra, Portugal

**Keywords:** Breast cancer, Glucose, Resistin, BMI, Age, Biomarker

## Abstract

**Background:**

The goal of this exploratory study was to develop and assess a prediction model which can potentially be used as a biomarker of breast cancer, based on anthropometric data and parameters which can be gathered in routine blood analysis.

**Methods:**

For each of the 166 participants several clinical features were observed or measured, including age, BMI, Glucose, Insulin, HOMA, Leptin, Adiponectin, Resistin and MCP-1. Machine learning algorithms (logistic regression, random forests, support vector machines) were implemented taking in as predictors different numbers of variables. The resulting models were assessed with a Monte Carlo Cross-Validation approach to determine 95% confidence intervals for the sensitivity, specificity and AUC of the models.

**Results:**

Support vector machines models using Glucose, Resistin, Age and BMI as predictors allowed predicting the presence of breast cancer in women with sensitivity ranging between 82 and 88% and specificity ranging between 85 and 90%. The 95% confidence interval for the AUC was [0.87, 0.91].

**Conclusions:**

These findings provide promising evidence that models combining age, BMI and metabolic parameters may be a powerful tool for a cheap and effective biomarker of breast cancer.

**Electronic supplementary material:**

The online version of this article (10.1186/s12885-017-3877-1) contains supplementary material, which is available to authorized users.

## Background

Breast cancer screening is an important strategy to allow for early detection and ensure a greater probability of having a good outcome in treatment. Robust predictive models based on data which may be collected in routine consultation and blood analysis are sought to provide an important contribution by offering more screening tools. In this work we aim to assess how models based on data which can be collected in routine blood analyses - notably, Glucose, Insulin, HOMA, Leptin, Adiponectin, Resistin, MCP-1, Age and Body Mass Index (BMI) - may be used to predict the presence of breast cancer. We believe that these parameters are a good set of candidates, as we recently verified a deregulation in their profile in obesity-associated breast cancer, [1].

Several candidates for biomarkers of breast cancer have been reported in the literature, [2]. In 2008 serum levels of tissue polypeptide-specific antigen, breast cancer-specific cancer antigen 15.3 (CA15–3), and insulin-like growth factor binding protein-3 (IGFBP-3) were introduced as predictors on a logistic regression. A subsequent receiver operating characteristic (ROC) analysis yielded an area under the ROC curve (AUC) value of 0.86, sensitivity 85% and specificity 62% when distinguishing controls from patients with breast cancer, [3]. BMI, Leptin, CA15–3 and the ratio between Leptin and Adiponectin used together were assessed as a biomarker for breast cancer in [4] (2013). Though very high values are presented for the specificity (80%) and the sensitivity (83.3%), the confidence intervals reported were [29.9%, 99.0%] and [36.5%, 99.1%], respectively. The lower bounds reported for the confidence intervals suggest that the prediction is not robust. Dalamaga et al. [5] assessed serum Resistin as a predictor of postmenopausal breast cancer and found an AUC value of 0.72, 95% CI [0.64, 0.79]. In 2015, a similar analysis was performed for Leptin, Resistin and Visfatin, [6]. The 95% confidence intervals for the AUC values found were [0.72, 0.87], [0.82, 0.93] and [0.64, 0.80], respectively. In terms of specificity and sensitivity, the values reported were 95.1 and 88.2% for leptin, 98.8 and 72.1% for Resistin and 97.6 and 92.6% for Visfatin. However, these values are inconsistent with the ROC curves plotted in the article, [7]. Also in 2015, serum Irisin levels were found to discriminate breast cancer patients with 62.7% sensitivity and 91.1% specificity, [8]. It is noteworthy that in the analysis of each of all articles mentioned in this paragraph, the data was not split into a training set and a test set. This implies that the models generated were assessed on the same data on which they were based, which is not necessarily a good indicator of performance on future data, [9]. In [10] the authors did indeed use a test set to evaluate potential biomarkers (promotor methylation of the tumour-suppressor genes SFRP1, SFRP2, SFRP5, ITIH5, WIF1, DKK3 and RASSF1A in cfDNA extracted from serum) for blood-based breast cancer screening. The sensitivity and specificity achieved using ITIH5, DKK3 and RASSF1A promoter methylation to distinguish between women with breast cancer and healthy controls was 67 and 69%, respectively, with the 95% confidence interval for the AUC being [0.63, 0.76].

Besides studies evaluating potential biomarkers for diagnosis, other authors have looked at breast cancer from other perspectives. In 2012 ten potential cancer serum biomarkers (Osteopontin, Haptoglobin, CA15–3, Carcinoembryonic Antigen, Cancer Antigen 125, Prolactin, Cancer Antigen 19–9, α-Fetoprotein, Leptin and Migration Inhibitory Factor) were studied to predict early stage breast cancer in samples collected before clinical diagnosis, but it was not possible to accurately differentiate samples from controls from those patients, [11]. In [12] a prediction model for breast cancer patients pathologic response before neoadjuvant chemotherapy was built and assessed. The predictors were tumour haemoglobin parameters measured by ultrasound-guided near-infrared optical tomography in conjunction with standard pathologic tumour characteristics. Several authors focused on assessing the risk of breast cancer, [13–15]. Finally, artificial intelligence and machine learning techniques were applied to databases made publicly available in the UCI Machine Learning Repository. In particular, there has been an extensive amount of work published on the Wisconsin Breast Cancer Dataset (WBCD), the Wisconsin Diagnosis Breast Cancer (WDBC) and the Wisconsin Prognosis Breast Cancer (WPBC), see for example [16–19]. In the same order, they provide cytology data which can be used for distinguishing malignant from benign samples, features computed from a digitized image of a fine needle aspirate of a breast mass again used for classifying as malignant or benign and follow-up data for breast cancer patients that can be used to predict cancer recurrence.

The models proposed in this work are based on a population with early-diagnosed breast cancer, whose extension to larger and more heterogeneous populations should subsequently be assessed. The description of the data collected and statistical methods used in the article are presented on the Methods section. The Results section is split into three subsections: first the characteristic features of the sample are described, then a univariate analysis is performed to assess the diagnostic value of each one of the nine aforementioned parameters and finally a multivariate analysis is performed wherein predictors are combined. The results are then discussed on a separate section and finally the main conclusions are presented.

## Methods

### Participants

Women newly diagnosed with breast cancer (BC) were recruited from the Gynaecology Department of the University Hospital Centre of Coimbra (CHUC) between 2009 and 2013. For each patient, the diagnosis came from a positive mammography and was histologically-confirmed. All samples were naïve, i.e., collected before surgery and treatment. All the patients with treatment before the consultation were excluded. Female healthy volunteers were selected and enrolled in the study as controls. All patients had had no prior cancer treatment and all participants were free from any infection or other acute diseases or comorbidities at the time of enrolment in the study. The latter was approved by the Ethical Committee of CHUC and all participants gave their written informed consent prior to entering the study. Further details of the patient study had been reported previously, [1]. The goal was then to assess hyperresistinemia and metabolic dysregulation in breast cancer. A total of 64 women with BC and 52 healthy volunteers was included in the present study - 38 participants that had been included in [1] were now excluded due to having BMI above 40 kg/m^2^ or due to the absence of at least one of the quantitative variables.

### Sample analysis

Blood samples were all collected at the same time of the day after an overnight fasting. Clinical, demographic and anthropometric data was collected for all participants, under similar conditions, always by the same research physician and during the first consultation. Collected data included age, weight, height and menopausal status (for each participant, this status expressed whether she was at least 12 months after menopause or reported a bilateral oophorectomy). The BMI, expressed in kg/m2, was determined dividing the weight by the squared height. Additionally, several measurements were extracted at the Laboratory of Physiology of the Faculty of Medicine of University of Coimbra from peripheral venous blood vials collected in the hospital for all participants. The fasting blood was first centrifuged (2500 g) at 4 °C and stored at −80 °C for biochemical determinations as previously described in [1]. Briefly, Serum Glucose levels were determined by an automatic analyser using a commercial kit (Olympus - Diagnóstica Portugal, Produtos de Diagnóstico SA, Portugal). Serum values of Leptin, Adiponectin and Resistin and the Chemokine Monocyte Chemoattractant Protein 1 (MCP-1) were assessed using the following commercial enzyme-linked immunosorbent assay kits: Duo Set ELISA Development System Human Leptin, Duo Set ELISA Development System Human Adiponectin, Duo Set ELISA Development System Human Resistin, all from R&D System, UK, and Human MCP-1 ELISA Set, BD Biosciences Pharmingen, CA, EUA. Plasma levels of Insulin were also measured by ELISA kit using Mercodia Insulin ELISA, Mercodia AB, Sweden. Homeostasis Model Assessment (HOMA) index was calculated to evaluate insulin resistance: [HOMA = logarithm ((If) x (Gf)) / 22.5, where (If) is the fasting insulin level (μU/mL) and (Gf) is the fasting Glucose level (mmol/L)]. Finally, for BC patients, tumour tissue was obtained by mastectomy or tumourectomy. Tumour type, grade and size and lymph node involvement were evaluated by a trained pathologist at the Anatomic Pathology Department of CHUC. For cancer staging notation, the TNM classification of malignant tumours was used. The status of Estrogen and Progesterone receptors and HER-2 protein was evaluated by immunohistochemistry following routine diagnostic techniques. When the results were ambiguous for HER-2 protein, the confirmation was made by FISH/SISH technique.

### Statistical analysis

A univariate statistical analysis was initially performed wherein each quantitative variable was assessed for normality, both for controls and patients, using Shapiro-Wilk tests. Since the normality assumptions were not met, median values and interquartile ranges were computed for each variable, which was then further compared between groups using Mann-Whitney U tests. Categorical variables were described in terms of absolute frequencies and percentages. The menopausal status of controls and patients was assessed through a simple cross-tabulation and by using the chi-square test. Finally, a ROC analysis was performed for each of the nine parameters (Age, BMI, Glucose, Insulin, HOMA, Leptin, Adiponectin, Resistin and MCP-1). The area under the ROC curve was computed as an indicator of the diagnostic predictive value associated to each variable, [20]. For each of the latter with a AUC value greater than 0.5, the pair of sensitivity and specificity values that maximise the Youden Index were computed, [21].

**Fig. 1:**
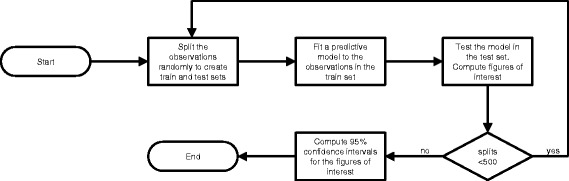
Flowchart of the computer routine for assessing the performance of each classification method when applied to *n* features

A preliminary step for the multivariate analysis consisted of determining the importance as breast cancer predictors of each of the variables for which a ROC analysis had been performed. This was done by using the Gini coefficient to measure the total decrease in node impurities associated to splitting on the variable in a Random Forest algorithm, averaged over all trees, [22]. Predictive models were then built with three classification algorithms: logistic regression (LR), support vector machines (SVM) and random forests (RF). Each model took in as predictors the *n* variables that had been found to be the most important predictors. Different values for *n* were tested, from *n* = 2 to *n* = 6 and also taking *n* = 9 to include all variables as predictors. A Monte Carlo Cross-Validation (MCCV) approach was adopted, wherein LR, SVM and RF models were built on a training set and assessed in terms of three figures of interest attained on a test set: the AUC resulting from a ROC analysis, the specificity and the sensitivity, see Fig. 1, [23]. The training set corresponded to 69.8% of the total amount of data (45 out of 62 patients and 36 out of 52 controls). By further repeating a total of 500 times the process where data is randomly assigned to the training and test sets and models are build and assessed, 95% confidence intervals were computed for each figure of interest from the empirical percentiles, as in [24].

A power analysis was conducted following the approach described in [25] with a few adaptations: the large artificial data set consisted of a 20 fold replication of subjects, simulations were performed for MCCV, 500 random splits of the data were considered, 100 iterations were performed for each sample size and the models were considered to be reliable when the absolute distance between the AUC computed over the development and validation sets was a value up to 0.1.

The univariate statistical analysis of the data was performed using the IBM® SPSS® version 21 for Windows. The multivariate analysis was done using algorithms implemented in R (R 3.0.2) and several packages from https://cran.r-project.org/. The scripts with the algorithm implementation are made available as Additional file 1. The level of significance adopted was α = 0.05.

## Results

This section is split into three subsections. In the first the clinical features (age and metabolic and inflammatory profile) of patients are compared with those of healthy controls. The group of patients is further described in terms of their tumour anatomopathological characteristics. A univariate analysis assessing the individual diagnosis values of several parameters is described in the second subsection. The final subsection describes a multivariate analysis wherein several parameters were combined and models to distinguish between healthy subjects and those with breast cancer were generated and assessed.

### Sample characteristics

**Fig. 2:**
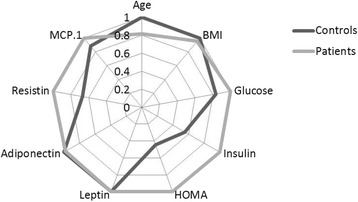
Profiles of the clinical features of features of patients with breast cancer (*n* = 64) and healthy controls (*n* = 52). BMI - body mass index; MCP-1 - monocyte chemoattractant protein-1, HOMA - homeostasis model assessment for insulin resistance

The quantitative features of patients and healthy controls are described in terms of their medians and interquartile ranges in Table 1. They are also represented graphically in the radial chart in Fig. 2 - each radial line corresponds to one variable, the dark grey line corresponds to controls and the light grey line to patients. The values represented, for each group and variable, are the median values divided by the maximum median value attained for that variable by any of the groups.

**Table 1:** Descriptive statistics of the clinical features (notably, age, BMI and inflammatory and metabolic parameters) of the 64 patients with breast cancer and 52 healthy controls^a^

	Patients	Controls	*p*-value
Age (years)	53.0 (23.0)	65.0 (33.2)	0.479
BMI (kg/m^2^)	27.0 (4.6)	28.3 (5.4)	0.202
Glucose (mg/dL)	105.6 (26.6)	88.2 (10.2)	<0.001
Insulin (μU/mL)	12.5 (12.3)	6.9 (4.9)	0.027
HOMA	3.6 (4.6)	1.6 (1.2)	0.003
Leptin (ng/mL)	26.6 (19.2)	26.6 (19.3)	0.949
Adiponectin (μg/mL)	10.1 (6.2)	10.3 (7.6)	0.767
Resistin (ng/mL)	17.3 (12.6)	11.6 (11.4)	0.002
MCP-1(pg/dL)	563.0 (384.0)	499.7 (292.2)	0.504

^a^Values are given as median (interquartile range). The *p*-values included in the table were obtained with Mann-Whitney U tests, after normality assumptions were assessed, for each variable, with a Shapiro-Wilk test. *BMI* body mass index, *MCP-1* monocyte chemoattractant protein-1, *HOMA* homeostasis model assessment for insulin resistance

In spite of the median age varying noticeably between controls and patients, no statistical differences for age (*p* = 0.479) medians were found between the two groups of participants. The same holds for or BMI (*p* = 0.272). It is worth adding that the mean ages are similar - 58.1 and 56.7 years, respectively in controls and patients - and the age ranges from 24 to 89 in controls and from 34 to 86 in patients. In terms of the metabolic parameters collected, statistically significant differences can be found in terms of Glucose, Insulin, HOMA and Resistin, all of which are higher for the patients. Leptin, Adiponectin and MCP-1 values were found to be similar between the two groups. The menopausal status was also compared between the groups - 38 of the 64 (59%) patients and 33 of the 52 (63%) of the controls were found to be post-menopausal - the difference between the proportions of post-menopausal women in the two groups was found not to be statistically significant, χ^2^(1, *N* = 116) = 0.202, *p* = 0.653.

The anatomopathological characteristics of breast tumour are included in Table 2.

**Table 2:** Anatomopathological characteristics for patients with breast cancer^a^

Tumor grade	Tumor stage	Tumor size	Lymph node involvement	ER, PR, CERB2
I- 13 (21.3%)	0–5 (7.8%)	≤2 cm- 54 (84.4%)	Yes- 27 (42.2%)	ER+ 53 (82.8%)
II- 39 (63.9%)	I- 29 (45.3%)	>2 cm- 10 (15.6%)	No- 37 (57.8%)	ER- 5 (7.8%)
III- 9 (14.8%)	II- 30 (46.9%)			
				PR+ 52 (81.3%) PR- 6 (9.4%) CERB2+ 18 (28.1%) CERB2–40 (62.5%)

^a^Values for qualitative variables are given as counts (percentages). The last column corresponds to the status of oestrogen (ER) and progesterone (PR) receptors and protein CerbB2

### Univariate analysis

A ROC analysis was performed for each potential biomarker. Confidence intervals at a 95% confidence level were found for the corresponding AUC values, see Table 3. The sensitivity and specificity values that maximise the Youden Index were also computed for four of the variables for which some diagnosis value was found. Glucose was the parameter for which higher sensitivity was attained (77%), though the specificity was low (67%). The other three variables, Insulin, HOMA and Resistin, were found to be more specific (specificity ranging between 79 and 85%) than sensitive (sensitivity ranging between 47 and 55%).

**Table 3:** Univariate analysis of how well each parameter allows distinguishing between patients with BC and controls^a^

Variables	95% CI for AUC	Sensitivity	Specificity
Age	[0.42, 0.64]	–	–
BMI	[0.46, 0.68]	–	–
Glucose	[0.68, 0.85]	77	67
Insulin	[0.52, 0.72]	47	83
HOMA	[0.56, 0.76]	50	85
Leptin	[0.39, 0.60]	–	–
Adiponectin	[0.41, 0.62]	–	–
Resistin	[0.57, 0.77]	55	79
MCP-1	[0.36, 0.57]	–	–

^a^A ROC analysis performed for each variable. The resulting 95% confidence intervals for the AUC were computed. For variables for which the confidence interval did not contain the number 0.5, the sensitivity and specificity that maximise Youden Index were computed

### Multivariate analysis

The Gini coefficient was used to obtain an a priori estimate for how much the variables being assessed as biomarkers can bring to a predictive model of the presence of breast cancer. By decreasing order of importance, the variables were: Glucose, Resistin, Age, BMI, HOMA, Leptin, Insulin, Adiponectin, MCP-1.

**Fig. 3:**
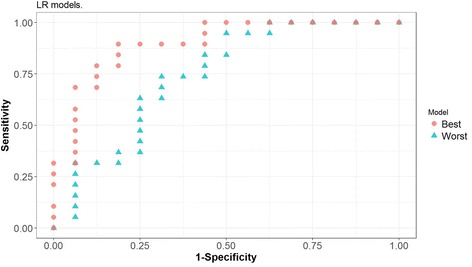
ROC curves corresponding to the best and worst Logistic Regression (LR) models generated with four predictors in the cross-validation procedure

**Fig. 4:**
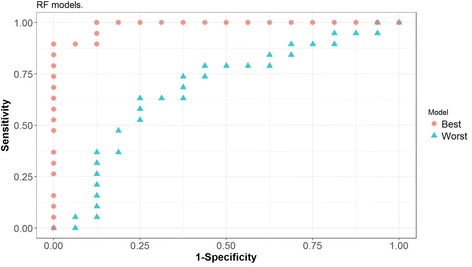
ROC curves corresponding to the best and worst Random Forest (RF) models generated with four predictors in the cross-validation procedure

**Fig. 5:**
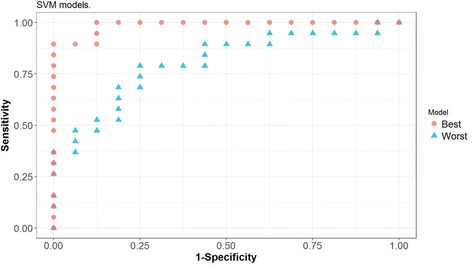
ROC curves corresponding to the best and worst Support Vector Machine (SVM) models generated with four predictors in the cross-validation procedure

Models were built over training sets using different classification methods: LR, RF and SVM. Different combinations of variables used as predictors were tested. Confidence intervals at a 95% level of confidence were computed in the test set for the AUC, sensitivity and specificity values obtained for the models, see Table 4. The best combination of sensitivity - 95% CI [82.2%, 87.5%] - and specificity - 95% CI [84.5%, 89.7%] - is achieved using SVM with 4 predictors, notably Glucose, Resistin, Age and BMI. The corresponding 95% confidence interval for the AUC is [0.866, 0.905], which can be interpreted as the model having a very good capacity to distinguish between patients and controls based on the 4 predictors. For each classifier (LR, RF and SVM), ROC curves were obtained for both the best and the worst models (in terms of having attained the lowest and highest AUC value, respectively) out of the 500 models obtained in the cross-validation procedure, see Figs. 3, 4 and 5.

**Table 4:** Multivariate analysis of how well the parameters allow distinguishing between patients with BC and controls^a^

Variables	Figures of interest	Classifier		
		LR	RF	SVM
V1-V2	AUC	[0.76, 0.81]	[0.70, 0.75]	[0.76, 0.81]
	Sensitivity	[0.75, 0.81]	[0.75, 0.82]	[0.81, 0.86]
	Specificity	[0.73, 0.80]	[0.63, 0.70]	[0.70, 0.76]
V1-V3	AUC	[0.76, 0.80]	[0.81, 0.85]	[0.82, 0.86]
	Sensitivity	[0.74, 0.81]	[0.85, 0.90]	[0.87, 0.92]
	Specificity	[0.74, 0.80]	[0.72, 0.78]	[0.78, 0.83]
V1-V4	AUC	[0.79, 0.83]	[0.84, 0.88]	[0.87, 0.91]
	Sensitivity	[0.72, 0.78]	[0.80, 0.86]	[0.82, 0.88]
	Specificity	[0.80, 0.87]	[0.81, 0.87]	[0.84, 0.90]
V1-V5	AUC	[0.79, 0.83]	[0.82, 0.87]	[0.86, 0.90]
	Sensitivity	[0.73, 0.79]	[0.79, 0.85]	[0.84, 0.90]
	Specificity	[0.81, 0.87]	[0.77, 0.83]	[0.81, 0.87]
V1-V6	AUC	[0.78, 0.83]	[0.82, 0.86]	[0.83, 0.88]
	Sensitivity	[0.74, 0.80]	[0.79, 0.85]	[0.81, 0.86]
	Specificity	[0.79, 0.85]	[0.76, 0.82]	[0.80, 0.86]
V1-V9	AUC	[0.76, 0.81]	[0.78, 0.83]	[0.81, 0.85]
	Sensitivity	[0.70, 0.76]	[0.78, 0.85]	[0.75, 0.81]
	Specificity	[0.80, 0.86]	[0.70, 0.77]	[0.78, 0.84]

^a^For each classifier (*LR* logistic regression, *RF* random forest, *SVM* support vector machine), predictive models were created taking in as predictors the variables deemed more significant. The predictive capacity of each model was computed resorting to a ROC analysis and determining the pair of values of specificity and sensitivity that maximise the Youden index. Again for each model, the resulting AUC value depends on the number of variables included, as can be seen on the table below, where V1 = Glucose, V2 = Resistin, V3 = Age, V4 = BMI - body mass index, V5 = HOMA - homeostasis model assessment for insulin resistance, V6 = Leptin, V7 = Insulin, V8 = Adiponectin, V9 = MCP-1 - monocyte chemoattractant protein-1

### Power analysis

The sample size required for the power to be at least 80% for all the modelling techniques used was found to have to be nearly 15 times greater than the number of predictors for the Monte Carlo Cross-Validation procedure to attain reliable results. The number of subjects included in the study was 116 (64 patients and 52 controls) and the number of predictors used in models ranged from 2 to 9.

## Discussion

In this study we propose a model for breast cancer detection based on biomarkers. The putative biomarkers assessed were Glucose, Resistin, Age, BMI, HOMA, Leptin, Insulin, Adiponectin, MCP-1. Using solely the combination of the first 4 variables on a predictive model using support vector machines allowed achieving the following 95% confidence intervals for sensitivity and specificity on a test set: [82%, 88%] and [84%, 90%], respectively. Additionally, the confidence interval for the AUC was [0.87, 0.91]. The intention is not to propose these models as an alternative to digital mammography, which a large study showed to have a sensitivity of 41% and a specificity of 98% at detecting which women would present breast cancer within 455 days of study entry and 70% sensitivity and 92% specificity when the follow up was reduced to 365 days [26]. Rather, as it is a rather noninvasive and inexpensive test which can be easily implemented in routine analysis by further measuring resistin (commercial kits allowing for the measurement of resistin are already available for under 20 euros per sample) and which we believe merits further study.

Previous studies had reported studying the diagnostic value for breast cancer of individual candidates for biomarkers [5, 6, 8] or combinations of candidates [3, 4, 10]. In [5], the 95% confidence interval for the AUC value found (over the whole set of data) for Resistin was [0.64, 0.79], which is consistent with that found on the present study, [0.57, 0.77]. The evidence found in [6] lacks clarification, [7]. The sensitivity and specificity values for serum Irisin levels found in [8] (again over the whole set of data) were 63 and 91%, respectively. The present study did not collect data on Irisin or Visfatin levels, which would be interesting to further include in a future study. Out of the studies using a combination of putative biomarkers for diagnosis purpose, only in [10] did the authors use a separate data set to perform the assessment, as is good practice, [9]. The sensitivity and specificity values therein achieved were 67 and 69%, respectively, which fall below the predictive value found in the present article. In [3] a very good AUC value of 0.86 was found over the whole data set when using polypeptide-specific antigen, CA15–3, and IGFBP-3 as predictors, with sensitivity 85% and specificity 62%. The confidence intervals obtained in [4] are too wide for useful information to be drawn from the study.

The small sample size of the study is a limitation, as over-fitting is hard to avoid and may lead to artificially high accuracy results. We adopted a cross-validation procedure (notably, MCCV) to minimize bias, but it is not possible to fully eliminate it. Accordingly, a power analysis was performed (where differences in AUC up to 0.1 were considered acceptable), suggesting that the sample size of the current study is sufficient for a MCCV technique with models with up to 6 predictors to be considered reliable for the sake of internal validation. However, that is not the case for models with 9 predictors, which should be interpreted with added caution. Moreover, adopting stricter constraints as in [25] (where differences in AUC greater than 0.01 are considered relevant for this purpose) implies increasing the sample size - the authors suggest that for a LR approach, 20 to 50 events per variable will have to be considered for an acceptable power to be attained. Note that this number was reached considering a split-sample approach, which behaves differently from MCCV.

In addition to considering increasing the sample size in the future, external validation should be sought, [27]. Also, the range and distribution of ages could benefit from being more similar between groups, though they are already quite close in average. It should also be noted that not all of the 116 participants in the study are in the age proposed by the 2015 American Cancer Society Guideline [28] for undergoing screening mammography, which is a limitation to be taken into account if the current model is adopted for screening purposes. Indeed, 27 participants (15 controls and 12 patients) were aged less than 45 years old, with 15 being younger than 40 years old. There were also 22 participants (15 controls and 7 patients) over the age for which the ACS proposes discontinuing screening.

Finally, we note that the focus of this work was not in optimising the accuracy of the classifiers, but rather assessing the predictive value of the set of predictors. Still, with the data used in this study to build the prediction models, it is possible to try to achieve better diagnosis accuracy. Notably, different classifiers or ensemble methods may be considered, the amount of data allocated to the training or test sets may be altered or data imputation techniques may be used to deal with cases that were excluded here due to missing data.

## Conclusions

Based on Resistin, Glucose, Age and BMI, the presence of breast cancer in women could be predicted on a test data set with sensitivity ranging between 82 and 88% and specificity ranging between 85 and 90% (95% CI for the AUC is [0.87, 0.91]). This suggests that Resistin and Glucose, taken together with Age and BMI, may be considered a good set of candidates for breast cancer biomarkers to implement into screening tests. As this procedure intends to increase the ease of diagnosis of breast cancer, it may potentially have great impact on the health of many women.

## Additional file

Additional file 1: R script with the algorithm implementation. (ZIP 330 kb)

## Abbreviations

AUC: Area under the curve
BC: Breast cancer
BMI: Body mass index
HOMA: Homeostasis Model Assessment
LR: Logistic regression
MCCV: Monte Carlo cross-validation
MCP-1: Chemokine Monocyte Chemoattractant Protein 1
RF: Random forest
ROC: Receiver operating characteristic
SVM: Support vector machine
