# Supplementary material for: Using Resistin, glucose, age and BMI to predict the presence of breast cancer
Source: BMC Cancer. 2018 Jan 4;18:29. doi: 10.1186/s12885-017-3877-1 (PMC5755302; doi:10.1186/s12885-017-3877-1)
Supplement: Additional file 1: — R script with the algorithm implementation. (ZIP 330 kb) [file 12885_2017_3877_MOESM1_ESM.zip › auxiliaryR2.pdf]

MAIN.RMD

---

title: "Can we predict breast cancer using age and metabolic parameters?"

author: Miguel Patrício(1), José Pereira(2), Joana Crisóstomo(3), Manuel Gomes(4), Raquel Seiça(2) and Francisco Caramelo(1)

output: pdf_document

graphics: yes

---

(1) Laboratory of Biostatistics and Medical Informatics and IBILI - Faculty of Medicine, University of Coimbra

(2) Faculty of Medicine, University of Coimbra

(3) Laboratory of Physiology, IBILI - Faculty of Medicine of University of Coimbra

(4) Department of Internal Medicine - University Hospital Centre of Coimbra

1. Short intro:

There's quantitative data (age+metabolic parameters) concerning two groups: controls and patients with cancer. The goal is to build predictive models, based on the data, to be able to tell who might have breast cancer.

2. About the data

```{r loading data, warning=FALSE, echo=FALSE, message=FALSE}
```

````
library(xlsx)

library(randomForest)

library(e1071)

library(pROC)

library(dplyr)

library(doBy)

library(ggplot2)

library(pscl)

library(ResourceSelection)

cancerdata=read.xlsx("cancer_data.xlsx", sheetIndex = 1)

levels(cancerdata$Classification)=c("control", "patient")

```
````

The first rows of the data look as follows:

````
```{r, warning=FALSE, echo=FALSE, message=FALSE}

head(cancerdata)

```
````

There are no missing values in the data. Overall, there are the following numbers of patients and healthy controls:

````
```{r, warning=FALSE, echo=FALSE, message=FALSE}

table(cancerdata$Classification)

```
````
````

For each variable, its median value and interquartile range, for each of the two aforementioned groups, are given by:

```{r, warning=FALSE, echo=FALSE, message=FALSE}

Agedata=summaryBy(Age ~ Classification, data=cancerdata, FUN=c(median,IQR))

Agedata=cbind(Agedata[1, 2:3], Agedata[2, 2:3], wilcox.test(Age ~ Classification, data=cancerdata)[3])

colnames(Agedata)=c("Control.median", "Control.IQR", "Patient.median", "Patient.IQR", "p-value")

BMIdata=summaryBy(BMI ~ Classification, data=cancerdata, FUN=c(mean,sd))

BMIdata=cbind(BMIdata[1, 2:3], BMIdata[2, 2:3], wilcox.test(BMI ~ Classification, data=cancerdata)[3])

colnames(BMIdata)=c("Control.median", "Control.IQR", "Patient.median", "Patient.IQR", "p-value")

Glucosedata=summaryBy(Glucose ~ Classification, data=cancerdata, FUN=c(mean,sd))

Glucosedata=cbind(Glucosedata[1, 2:3], Glucosedata[2, 2:3], wilcox.test(Glucose ~ Classification, data=cancerdata)[3])

colnames(Glucosedata)=c("Control.median", "Control.IQR", "Patient.median", "Patient.IQR", "p-value")

Insulindata=summaryBy(Insulin ~ Classification, data=cancerdata, FUN=c(mean,sd))

Insulindata=cbind(Insulindata[1, 2:3], Insulindata[2, 2:3], wilcox.test(Insulin ~ Classification, data=cancerdata)[3])

colnames(Insulindata)=c("Control.median", "Control.IQR", "Patient.median", "Patient.IQR", "p-value")

HOMAdata=summaryBy(HOMA ~ Classification, data=cancerdata, FUN=c(mean,sd))

HOMAdata=cbind(HOMAdata[1, 2:3], HOMAdata[2, 2:3], wilcox.test(HOMA ~ Classification, data=cancerdata)[3])
```

```
colnames(HOMAdata)=c("Control.median", "Control.IQR", "Patient.median", "Patient.IQR", "p-value")

Leptindata=summaryBy(Leptin ~ Classification, data=cancerdata, FUN=c(mean,sd))
Leptindata=cbind(Leptindata[1, 2:3], Leptindata[2, 2:3], wilcox.test(Leptin ~ Classification, data=cancerdata)[3])
colnames(Leptindata)=c("Control.median", "Control.IQR", "Patient.median", "Patient.IQR", "p-value")

Adiponectindata=summaryBy(Adiponectin ~ Classification, data=cancerdata, FUN=c(mean,sd))
Adiponectindata=cbind(Adiponectindata[1, 2:3], Adiponectindata[2, 2:3], wilcox.test(Adiponectin ~ Classification, data=cancerdata)[3])
colnames(Adiponectindata)=c("Control.median", "Control.IQR", "Patient.median", "Patient.IQR", "p-value")

Resistindata=summaryBy(Resistin ~ Classification, data=cancerdata, FUN=c(mean,sd))
Resistindata=cbind(Resistindata[1, 2:3], Resistindata[2, 2:3], wilcox.test(Resistin ~ Classification, data=cancerdata)[3])
colnames(Resistindata)=c("Control.median", "Control.IQR", "Patient.median", "Patient.IQR", "p-value")

MCP.1data=summaryBy(MCP.1 ~ Classification, data=cancerdata, FUN=c(mean,sd))
MCP.1data=cbind(MCP.1data[1, 2:3], MCP.1data[2, 2:3], wilcox.test(MCP.1 ~ Classification, data=cancerdata)[3])
colnames(MCP.1data)=c("Control.median", "Control.IQR", "Patient.median", "Patient.IQR", "p-value")

summarytable=rbind(Agedata, BMIdata, Glucosedata, Insulindata, HOMAdata, Leptindata, Adiponectindata, Resistindata, MCP.1data)
```

rownames(summarytable)=c("Age", "BMI", "Glucose", "Insulin", "HOMA", "Leptin", "Adiponectin", "Resistin", "MCP.1")

summarytable[, 1:4]=round(summarytable[, 1:4], digits=1)

summarytable[, 5]=round(summarytable[, 5], digits=4)

summarytable

```

The p-values included in the table were obtained with Mann-Whitney tests, after normality assumptions were assessed, for each variable, with a Shapiro-Wilk test:

```{r, warning=FALSE, echo=FALSE, message=FALSE}

x=cancerdata$Age

Agenormality=c(shapiro.test(subset(x, cancerdata$Classification=="control"))$p.value, shapiro.test(subset(x, cancerdata$Classification=="patient"))$p.value)

x=cancerdata$BMI

BMInormality=c(shapiro.test(subset(x, cancerdata$Classification=="control"))$p.value, shapiro.test(subset(x, cancerdata$Classification=="patient"))$p.value)

x=cancerdata$Glucose

Glucosenormality=c(shapiro.test(subset(x, cancerdata$Classification=="control"))$p.value, shapiro.test(subset(x, cancerdata$Classification=="patient"))$p.value)

x=cancerdata$Insulin

Insulinnormality=c(shapiro.test(subset(x, cancerdata$Classification=="control"))$p.value, shapiro.test(subset(x, cancerdata$Classification=="patient"))$p.value)

x=cancerdata$HOMA

```
HOMAnormality=c(shapiro.test(subset(x, cancerdata$Classification=="control"))$p.value, shapiro.test(subset(x, cancerdata$Classification=="patient"))$p.value)

x=cancerdata$Leptin

Leptinnormality=c(shapiro.test(subset(x, cancerdata$Classification=="control"))$p.value, shapiro.test(subset(x, cancerdata$Classification=="patient"))$p.value)

x=cancerdata$Adiponectin

Adiponectinnormality=c(shapiro.test(subset(x, cancerdata$Classification=="control"))$p.value, shapiro.test(subset(x, cancerdata$Classification=="patient"))$p.value)

x=cancerdata$Resistin

Resistinnormality=c(shapiro.test(subset(x, cancerdata$Classification=="control"))$p.value, shapiro.test(subset(x, cancerdata$Classification=="patient"))$p.value)

x=cancerdata$MCP.1

MCP.1normality=c(shapiro.test(subset(x, cancerdata$Classification=="control"))$p.value, shapiro.test(subset(x, cancerdata$Classification=="patient"))$p.value)

normalitytests=rbind(Agenormality, BMInormality, Glucosenormality, Insulinnormality, HOMAnormality, Leptinnormality, Adiponectinnormality, Resistinnormality, MCP.1normality)

colnames(normalitytests)=c("Control", "Patient")

normalitytests=round(normalitytests, digits=4)

rownames(normalitytests)=c("Age", "BMI", "Glucose", "Insulin", "HOMA", "Leptin", "Adiponectin", "Resistin", "MCP.1")

normalitytests

```

We also build up a logistic regression model:

````
```{r, warning=FALSE, echo=TRUE, message=FALSE}

levels(cancerdata$Classification)=c(1, 2)

glmmodel=glm(Classification~.,data=cancerdata,family='binomial')

summary(glmmodel)

```
````

What we have here are the beta-coefficients (means and standard errors, the z-values, the p-values) and the odds ratios+confidence intervals:

````
```{r, warning=FALSE, echo=FALSE, message=FALSE}

glmtable=cbind(coef(summary(glmmodel)), exp(coef(summary(glmmodel))[, 1]),
exp(confint(glmmodel)))

colnames(glmtable)[5]="Odds ratios"

round(glmtable, digits=3)

```
````

As for McFaden's pseudo R square and the result of Hosmer-Lemeshow goodness of fit test:

````
```{r, warning=FALSE, echo=FALSE, message=FALSE}

pR2(glmmodel)[4]

hoslem.test(as.numeric(cancerdata$Classification)-1, fitted(glmmodel), g=10)


```
````

3. Methods

Our goal is to build and assess models that allow predicting who has breast cancer based on the predictors (age and metabolic parameters) that were measured or observed. We will build up models, each based on

* one of three classification methods: logistic regression (LR), support vector machines (SVM) or random forests (RF)

* some (or all) of the predictors available

We will proceed as follows:

1) We will start by evaluating the role of each variable. They will be ordered from more to less meaningful.

2) We will be looking to build up and assess models, using the three classifiers, for different situations:

    * for when all predictors are considered

    * for when successively more predictors are considered, starting with only two predictors, adding predictors in order of importance

3 a) Evaluating the role of each variable

To evaluate the role of each variable we will use the total decrease in node impurities (measured by the Gini coefficient) from splitting on the variable in the RF algorithm, averaged over all trees. We

also perform a ROC analysis for each variable and compute the specificity and sensitivity values that maximise Youden's index.

3 b) Classification algorithm

Given: a classifier (LR, SVM or RF), a set of predictors, the true labels:

1) split the data (predictors and corresponding labels) into train and test sets

2) generate models over the train set (using the classifier)

3) evaluate models over test set, by computing

* AUC
* accuracy (for the cutoff that maximises Youden's index)
* specificity (for the cutoff that maximises Youden's index)
* sensitivity (for the cutoff that maximises Youden's index)
* positive predictive value (for the cutoff that maximises Youden's index)
* negative predictive value (for the cutoff that maximises Youden's index)

4) repeat steps 1) to 3) a great number of times and computing confidence intervals for the five indicators (AUC, specificity, sensitivity, PPV, NPV)

3 c) Functions

We use classifiers (LR, SVM, RF) to see how accurately one can predict whether someone has cancer. In this section we use five different functions that are described in the appendix at the end of the document:

````
```{r, warning=FALSE, echo=TRUE, message=FALSE}
source('splitter.R')
source('LRclassifier.R')
source('SVMclassifier.R')
source('RFclassifier.R')
source('evaluateclassifier.R')
```
````

What these functions do is:

* splitter.R: it randomly splits cases (i.e., subjects) into two groups: one, so-called training group, containing 70% of the subjects, will be used to build up models. The other, containing the remaining 30% of the subjects, will be the test group which we will use to evaluate the models.

* LRclassifier.R: Given the train data and the test data, it uses logistic regression to build a model with the train data. It then predicts the outcomes (diagnosis) of the subjects in the test data (output: for each subject, the probability that she has cancer)

* SVMclassifier.R: Given the train data and the test data, it uses support vector machines to build a model with the train data. It then predicts the outcomes (diagnosis) of the subjects in the test data (output: for each subject, the probability that she has cancer)

* RFclassifier.R: Given the train data and the test data, it uses a random forest algorithm to build a model with the train data. It then predicts the outcomes (diagnosis) of the subjects in the test data (output: for each subject, the probability that she has cancer)

* evaluateclassifier.R: each classifier outputs the probability that each element in the test set may belong to the group of women with cancer. This function computes the corresponding AUC, specificity, sensitivity, positive predictive value and negative predictive value

4. Results

4 a) Assessing the role of each variable

Starting from a Random Forest model built with all variables avaliable we will build a variable importance plot to check the relative importance of each variable in predicting the final outcome:

```{r,warning=FALSE, echo=TRUE, message=FALSE}

RFmodel <- randomForest(Classification ~ ., data = cancerdata)

#varImpPlot(RFmodel, type=2)

importance.table=varImpPlot(RFmodel, type=2)

importance.table

rownames(importance.table)[order(importance.table, decreasing = TRUE)]
```

```{r,warning=FALSE, echo=TRUE, message=FALSE}

real.diagnosis=cancerdata$Classification


predictor=cancerdata$Age

results=evaluateclassifier(predictor, cancerdata)

computed_AUC=results[[1]]
```

```
computed_AUC_lower_bound=results[[7]]

computed_AUC_upper_bound=results[[8]]

computed_sensitivity=results[[4]]

computed_specificity=results[[3]]

classifier_Age=c(computed_AUC, computed_AUC_lower_bound,
        computed_AUC_upper_bound,computed_sensitivity, computed_specificity)


predictor=cancerdata$BMI

results=evaluateclassifier(predictor, cancerdata)

computed_AUC=results[[1]]

computed_AUC_lower_bound=results[[7]]

computed_AUC_upper_bound=results[[8]]

computed_sensitivity=results[[4]]

computed_specificity=results[[3]]

classifier_BMI=c(computed_AUC, computed_AUC_lower_bound,
        computed_AUC_upper_bound,computed_sensitivity, computed_specificity)


predictor=cancerdata$Glucose

results=evaluateclassifier(predictor, cancerdata)

computed_AUC=results[[1]]

computed_AUC_lower_bound=results[[7]]

computed_AUC_upper_bound=results[[8]]

computed_sensitivity=results[[4]]

computed_specificity=results[[3]]

classifier_Glucose=c(computed_AUC, computed_AUC_lower_bound,
          computed_AUC_upper_bound,computed_sensitivity, computed_specificity)
```

```
predictor=cancerdata$Insulin

results=evaluateclassifier(predictor, cancerdata)

computed_AUC=results[[1]]

computed_AUC_lower_bound=results[[7]]

computed_AUC_upper_bound=results[[8]]

computed_sensitivity=results[[4]]

computed_specificity=results[[3]]

classifier_Insulin=c(computed_AUC, computed_AUC_lower_bound,

              computed_AUC_upper_bound,computed_sensitivity, computed_specificity)


predictor=cancerdata$HOMA

results=evaluateclassifier(predictor, cancerdata)

computed_AUC=results[[1]]

computed_AUC_lower_bound=results[[7]]

computed_AUC_upper_bound=results[[8]]

computed_sensitivity=results[[4]]

computed_specificity=results[[3]]

classifier_HOMA=c(computed_AUC, computed_AUC_lower_bound,

            computed_AUC_upper_bound,computed_sensitivity, computed_specificity)


predictor=cancerdata$Leptin

results=evaluateclassifier(predictor, cancerdata)

computed_AUC=results[[1]]

computed_AUC_lower_bound=results[[7]]

computed_AUC_upper_bound=results[[8]]

computed_sensitivity=results[[4]]

computed_specificity=results[[3]]
```

```
classifier_Leptin=c(computed_AUC, computed_AUC_lower_bound,
                computed_AUC_upper_bound,computed_sensitivity, computed_specificity)

predictor=cancerdata$Adiponectin
results=evaluateclassifier(predictor, cancerdata)
computed_AUC=results[[1]]
computed_AUC_lower_bound=results[[7]]
computed_AUC_upper_bound=results[[8]]
computed_sensitivity=results[[4]]
computed_specificity=results[[3]]
classifier_Adiponectin=c(computed_AUC, computed_AUC_lower_bound,
                    computed_AUC_upper_bound,computed_sensitivity, computed_specificity)

predictor=cancerdata$Resistin
results=evaluateclassifier(predictor, cancerdata)
computed_AUC=results[[1]]
computed_AUC_lower_bound=results[[7]]
computed_AUC_upper_bound=results[[8]]
computed_sensitivity=results[[4]]
computed_specificity=results[[3]]
classifier_Resistin=c(computed_AUC, computed_AUC_lower_bound,
                  computed_AUC_upper_bound,computed_sensitivity, computed_specificity)

predictor=cancerdata$MCP.1
results=evaluateclassifier(predictor, cancerdata)
computed_AUC=results[[1]]
computed_AUC_lower_bound=results[[7]]
```

computed_AUC_upper_bound=results[[8]]

computed_sensitivity=results[[4]]

computed_specificity=results[[3]]

classifier_MCP.1=c(computed_AUC, computed_AUC_lower_bound,

computed_AUC_upper_bound,computed_sensitivity, computed_specificity)

univariate_diagnostic=rbind(classifier_Age, classifier_BMI, classifier_Glucose, classifier_Insulin, classifier_HOMA, classifier_Leptin, classifier_Adiponectin, classifier_Resistin, classifier_MCP.1)

univariate_diagnostic=as.data.frame(univariate_diagnostic)

colnames(univariate_diagnostic)=c('AUC', 'AUC_lower_bound_CI', 'AUC_upper_bound_CI', 'sensitivity', 'specificity')

univariate_diagnostic

```

## 4 b) Building up and assessing models

### 4 b) i) Using all possible predictors

```{r, warning=FALSE, echo=TRUE, message=FALSE, cache=FALSE}

number.of.iterations=500

RF_AUC=matrix(NA, number.of.iterations, 1)

RF_acc=matrix(NA, number.of.iterations, 1)

RF_specificity=matrix(NA, number.of.iterations, 1)

RF_sensitivity=matrix(NA, number.of.iterations, 1)

RF_PPV=matrix(NA, number.of.iterations, 1)

```
RF_NPV=matrix(NA, number.of.iterations, 1)
#AUC, accuracy, specificity, sensitivity, PPV and NPV associated to applying RF

SVM_AUC=matrix(NA, number.of.iterations, 1)
SVM_acc=matrix(NA, number.of.iterations, 1)
SVM_specificity=matrix(NA, number.of.iterations, 1)
SVM_sensitivity=matrix(NA, number.of.iterations, 1)
SVM_PPV=matrix(NA, number.of.iterations, 1)
SVM_NPV=matrix(NA, number.of.iterations, 1)
#AUC, accuracy, specificity, sensitivity, PPV and NPV associated to applying SVM

LR_AUC=matrix(NA, number.of.iterations, 1)
LR_acc=matrix(NA, number.of.iterations, 1)
LR_specificity=matrix(NA, number.of.iterations, 1)
LR_sensitivity=matrix(NA, number.of.iterations, 1)
LR_PPV=matrix(NA, number.of.iterations, 1)
LR_NPV=matrix(NA, number.of.iterations, 1)
#AUC, accuracy, specificity, sensitivity, PPV and NPV associated to applying LR

for (iterationindex in 1:number.of.iterations){

set.seed(iterationindex) # controls the random process of splitting the data
splitteddata=splitter(cancerdata, 0.7) # splits the data
trainset=splitteddata[[1]] # train set
testset=splitteddata[[2]] # test set
```

```
RF.prediction=RFclassifier(trainset, testset)
SVM.prediction=SVMclassifier(trainset, testset)
LR.prediction=LRclassifier(trainset, testset)

RF.evaluation=evaluateclassifier(RF.prediction, testset)
SVM.evaluation=evaluateclassifier(SVM.prediction, testset)
LR.evaluation=evaluateclassifier(LR.prediction, testset)


RF_AUC[iterationindex]=RF.evaluation[[1]]
RF_acc[iterationindex]=RF.evaluation[[2]]
RF_specificity[iterationindex]=RF.evaluation[[3]]
RF_sensitivity[iterationindex]=RF.evaluation[[4]]
RF_PPV[iterationindex]=RF.evaluation[[5]]
RF_NPV[iterationindex]=RF.evaluation[[6]]

SVM_AUC[iterationindex]=SVM.evaluation[[1]]
SVM_acc[iterationindex]=SVM.evaluation[[2]]
SVM_specificity[iterationindex]=SVM.evaluation[[3]]
SVM_sensitivity[iterationindex]=SVM.evaluation[[4]]
SVM_PPV[iterationindex]=SVM.evaluation[[5]]
SVM_NPV[iterationindex]=SVM.evaluation[[6]]

LR_AUC[iterationindex]=LR.evaluation[[1]]
LR_acc[iterationindex]=LR.evaluation[[2]]
LR_specificity[iterationindex]=LR.evaluation[[3]]
```

LR_sensitivity[iterationindex]=LR.evaluation[[4]]

LR_PPV[iterationindex]=LR.evaluation[[5]]

LR_NPV[iterationindex]=LR.evaluation[[6]]

}

```

So what we see that we obtain is

* for LR, the 95% confidence interval for the

  * AUC is [`r mean(LR_AUC)-qnorm(0.975)*sqrt(sd(LR_AUC)/number.of.iterations)`, `r mean(LR_AUC)+qnorm(0.975)*sqrt(sd(LR_AUC)/number.of.iterations)`]

  * accuracy is [`r mean(LR_acc)-qnorm(0.975)*sqrt(sd(LR_acc)/number.of.iterations)`, `r mean(LR_acc)+qnorm(0.975)*sqrt(sd(LR_acc)/number.of.iterations)`]

  * sensitivity is [`r mean(LR_sensitivity)-qnorm(0.975)*sqrt(sd(LR_sensitivity)/number.of.iterations)`, `r mean(LR_sensitivity)+qnorm(0.975)*sqrt(sd(LR_sensitivity)/number.of.iterations)`]

  * specificity is [`r mean(LR_specificity)-qnorm(0.975)*sqrt(sd(LR_specificity)/number.of.iterations)`, `r mean(LR_specificity)+qnorm(0.975)*sqrt(sd(LR_specificity)/number.of.iterations)`]

  * PPV is [`r mean(LR_PPV)-qnorm(0.975)*sqrt(sd(LR_PPV)/number.of.iterations)`, `r mean(LR_PPV)+qnorm(0.975)*sqrt(sd(LR_PPV)/number.of.iterations)`]
```

* NPV is [`r mean(LR_NPV)-qnorm(0.975)*sqrt(sd(LR_NPV)/number.of.iterations)`, `r mean(LR_NPV)+qnorm(0.975)*sqrt(sd(LR_NPV)/number.of.iterations)`]

* for RF, the 95% confidence interval for the

    * AUC is [`r mean(RF_AUC)-qnorm(0.975)*sqrt(sd(RF_AUC)/number.of.iterations)`, `r mean(RF_AUC)+qnorm(0.975)*sqrt(sd(RF_AUC)/number.of.iterations)`]

    * accuracy is [`r mean(RF_acc)-qnorm(0.975)*sqrt(sd(RF_acc)/number.of.iterations)`, `r mean(RF_acc)+qnorm(0.975)*sqrt(sd(RF_acc)/number.of.iterations)`]

    * sensitivity is [`r mean(RF_sensitivity)-qnorm(0.975)*sqrt(sd(RF_sensitivity)/number.of.iterations)`, `r mean(RF_sensitivity)+qnorm(0.975)*sqrt(sd(RF_sensitivity)/number.of.iterations)`]

    * specificity is [`r mean(RF_specificity)-qnorm(0.975)*sqrt(sd(RF_specificity)/number.of.iterations)`, `r mean(RF_specificity)+qnorm(0.975)*sqrt(sd(RF_specificity)/number.of.iterations)`]

    * PPV is [`r mean(RF_PPV)-qnorm(0.975)*sqrt(sd(RF_PPV)/number.of.iterations)`, `r mean(RF_PPV)+qnorm(0.975)*sqrt(sd(RF_PPV)/number.of.iterations)`]

    * NPV is [`r mean(RF_NPV)-qnorm(0.975)*sqrt(sd(RF_NPV)/number.of.iterations)`, `r mean(RF_NPV)+qnorm(0.975)*sqrt(sd(RF_NPV)/number.of.iterations)`]

* for SVM, the 95% confidence interval for the

* AUC is [`r mean(SVM_AUC)-qnorm(0.975)*sqrt(sd(SVM_AUC)/number.of.iterations)`, `r mean(SVM_AUC)+qnorm(0.975)*sqrt(sd(SVM_AUC)/number.of.iterations)`]

* accuracy is [`r mean(SVM_acc)-qnorm(0.975)*sqrt(sd(SVM_acc)/number.of.iterations)`, `r mean(SVM_acc)+qnorm(0.975)*sqrt(sd(SVM_acc)/number.of.iterations)`]

* sensitivity is [`r mean(SVM_sensitivity)-qnorm(0.975)*sqrt(sd(SVM_sensitivity)/number.of.iterations)`, `r mean(SVM_sensitivity)+qnorm(0.975)*sqrt(sd(SVM_sensitivity)/number.of.iterations)`]

* specificity is [`r mean(SVM_specificity)-qnorm(0.975)*sqrt(sd(SVM_specificity)/number.of.iterations)`, `r mean(SVM_specificity)+qnorm(0.975)*sqrt(sd(SVM_specificity)/number.of.iterations)`]

* PPV is [`r mean(SVM_PPV)-qnorm(0.975)*sqrt(sd(SVM_PPV)/number.of.iterations)`, `r mean(SVM_PPV)+qnorm(0.975)*sqrt(sd(SVM_PPV)/number.of.iterations)`]

* NPV is [`r mean(SVM_NPV)-qnorm(0.975)*sqrt(sd(SVM_NPV)/number.of.iterations)`, `r mean(SVM_NPV)+qnorm(0.975)*sqrt(sd(SVM_NPV)/number.of.iterations)`]

4 b) ii) Using only the most important predictor

As we can see, the benchmark results already have good accuracy in predicting the diagnosis. We will now see if we can do some feature engineering on the model variables in order to improve the quality of the model.

4 b) ii) Using the most important predictor

```{r,warning=FALSE, echo=TRUE, message=FALSE}
cancerdata_subset <- dplyr::select(cancerdata, Glucose, Classification)
```

```{r, warning=FALSE, echo=TRUE, message=FALSE, cache=FALSE}
number.of.iterations=500
RF_AUC=matrix(NA, number.of.iterations, 1)
RF_acc=matrix(NA, number.of.iterations, 1)
RF_specificity=matrix(NA, number.of.iterations, 1)
RF_sensitivity=matrix(NA, number.of.iterations, 1)
RF_PPV=matrix(NA, number.of.iterations, 1)
RF_NPV=matrix(NA, number.of.iterations, 1)
#AUC, accuracy, specificity, sensitivity, PPV and NPV associated to applying RF

SVM_AUC=matrix(NA, number.of.iterations, 1)
SVM_acc=matrix(NA, number.of.iterations, 1)
SVM_specificity=matrix(NA, number.of.iterations, 1)
SVM_sensitivity=matrix(NA, number.of.iterations, 1)
SVM_PPV=matrix(NA, number.of.iterations, 1)
SVM_NPV=matrix(NA, number.of.iterations, 1)
#AUC, accuracy, specificity, sensitivity, PPV and NPV associated to applying SVM

LR_AUC=matrix(NA, number.of.iterations, 1)
LR_acc=matrix(NA, number.of.iterations, 1)
LR_specificity=matrix(NA, number.of.iterations, 1)
LR_sensitivity=matrix(NA, number.of.iterations, 1)
```

```
LR_PPV=matrix(NA, number.of.iterations, 1)

LR_NPV=matrix(NA, number.of.iterations, 1)

#AUC, accuracy, specificity, sensitivity, PPV and NPV associated to applying LR




for (iterationindex in 1:number.of.iterations){


set.seed(iterationindex) # controls the random process of splitting the data

splitteddata=splitter(cancerdata_subset, 0.7) # splits the data

trainset=splitteddata[[1]] # train set

testset=splitteddata[[2]] # test set


RF.prediction=RFclassifier(trainset, testset)

SVM.prediction=SVMclassifier(trainset, testset)

LR.prediction=LRclassifier(trainset, testset)


RF.evaluation=evaluateclassifier(RF.prediction, testset)

SVM.evaluation=evaluateclassifier(SVM.prediction, testset)

LR.evaluation=evaluateclassifier(LR.prediction, testset)



RF_AUC[iterationindex]=RF.evaluation[[1]]

RF_acc[iterationindex]=RF.evaluation[[2]]

RF_specificity[iterationindex]=RF.evaluation[[3]]

RF_sensitivity[iterationindex]=RF.evaluation[[4]]

RF_PPV[iterationindex]=RF.evaluation[[5]]
```

```
RF_NPV[iterationindex]=RF.evaluation[[6]]

SVM_AUC[iterationindex]=SVM.evaluation[[1]]
SVM_acc[iterationindex]=SVM.evaluation[[2]]
SVM_specificity[iterationindex]=SVM.evaluation[[3]]
SVM_sensitivity[iterationindex]=SVM.evaluation[[4]]
SVM_PPV[iterationindex]=SVM.evaluation[[5]]
SVM_NPV[iterationindex]=SVM.evaluation[[6]]

LR_AUC[iterationindex]=LR.evaluation[[1]]
LR_acc[iterationindex]=LR.evaluation[[2]]
LR_specificity[iterationindex]=LR.evaluation[[3]]
LR_sensitivity[iterationindex]=LR.evaluation[[4]]
LR_PPV[iterationindex]=LR.evaluation[[5]]
LR_NPV[iterationindex]=LR.evaluation[[6]]

}
```

So what we see that we obtain is

* for LR, the 95% confidence interval for the

    * AUC is [`r mean(LR_AUC)-qnorm(0.975)*sqrt(sd(LR_AUC)/number.of.iterations)`, `r mean(LR_AUC)+qnorm(0.975)*sqrt(sd(LR_AUC)/number.of.iterations)`]

* accuracy is [`r mean(LR_acc)-qnorm(0.975)*sqrt(sd(LR_acc)/number.of.iterations)`, `r mean(LR_acc)+qnorm(0.975)*sqrt(sd(LR_acc)/number.of.iterations)`]

* sensitivity is [`r mean(LR_sensitivity)-qnorm(0.975)*sqrt(sd(LR_sensitivity)/number.of.iterations)`, `r mean(LR_sensitivity)+qnorm(0.975)*sqrt(sd(LR_sensitivity)/number.of.iterations)`]

* specificity is [`r mean(LR_specificity)-qnorm(0.975)*sqrt(sd(LR_specificity)/number.of.iterations)`, `r mean(LR_specificity)+qnorm(0.975)*sqrt(sd(LR_specificity)/number.of.iterations)`]

* PPV is [`r mean(LR_PPV)-qnorm(0.975)*sqrt(sd(LR_PPV)/number.of.iterations)`, `r mean(LR_PPV)+qnorm(0.975)*sqrt(sd(LR_PPV)/number.of.iterations)`]

* NPV is [`r mean(LR_NPV)-qnorm(0.975)*sqrt(sd(LR_NPV)/number.of.iterations)`, `r mean(LR_NPV)+qnorm(0.975)*sqrt(sd(LR_NPV)/number.of.iterations)`]

* for RF, the 95% confidence interval for the

* AUC is [`r mean(RF_AUC)-qnorm(0.975)*sqrt(sd(RF_AUC)/number.of.iterations)`, `r mean(RF_AUC)+qnorm(0.975)*sqrt(sd(RF_AUC)/number.of.iterations)`]

* accuracy is [`r mean(RF_acc)-qnorm(0.975)*sqrt(sd(RF_acc)/number.of.iterations)`, `r mean(RF_acc)+qnorm(0.975)*sqrt(sd(RF_acc)/number.of.iterations)`]

* sensitivity is [`r mean(RF_sensitivity)-qnorm(0.975)*sqrt(sd(RF_sensitivity)/number.of.iterations)`, `r mean(RF_sensitivity)+qnorm(0.975)*sqrt(sd(RF_sensitivity)/number.of.iterations)`]

* specificity is [`r mean(RF_specificity)-qnorm(0.975)*sqrt(sd(RF_specificity)/number.of.iterations)`, `r mean(RF_specificity)+qnorm(0.975)*sqrt(sd(RF_specificity)/number.of.iterations)`]

* PPV is [`r mean(RF_PPV)-qnorm(0.975)*sqrt(sd(RF_PPV)/number.of.iterations)`, `r mean(RF_PPV)+qnorm(0.975)*sqrt(sd(RF_PPV)/number.of.iterations)`]

* NPV is [`r mean(RF_NPV)-qnorm(0.975)*sqrt(sd(RF_NPV)/number.of.iterations)`, `r mean(RF_NPV)+qnorm(0.975)*sqrt(sd(RF_NPV)/number.of.iterations)`]

* for SVM, the 95% confidence interval for the

* AUC is [`r mean(SVM_AUC)-qnorm(0.975)*sqrt(sd(SVM_AUC)/number.of.iterations)`, `r mean(SVM_AUC)+qnorm(0.975)*sqrt(sd(SVM_AUC)/number.of.iterations)`]

* accuracy is [`r mean(SVM_acc)-qnorm(0.975)*sqrt(sd(SVM_acc)/number.of.iterations)`, `r mean(SVM_acc)+qnorm(0.975)*sqrt(sd(SVM_acc)/number.of.iterations)`]

* sensitivity is [`r mean(SVM_sensitivity)-qnorm(0.975)*sqrt(sd(SVM_sensitivity)/number.of.iterations)`, `r mean(SVM_sensitivity)+qnorm(0.975)*sqrt(sd(SVM_sensitivity)/number.of.iterations)`]

* specificity is [`r mean(SVM_specificity)-qnorm(0.975)*sqrt(sd(SVM_specificity)/number.of.iterations)`, `r mean(SVM_specificity)+qnorm(0.975)*sqrt(sd(SVM_specificity)/number.of.iterations)`]

* PPV is [`r mean(SVM_PPV)-qnorm(0.975)*sqrt(sd(SVM_PPV)/number.of.iterations)`, `r mean(SVM_PPV)+qnorm(0.975)*sqrt(sd(SVM_PPV)/number.of.iterations)`]

* NPV is [`r mean(SVM_NPV)-qnorm(0.975)*sqrt(sd(SVM_NPV)/number.of.iterations)`, `r mean(SVM_NPV)+qnorm(0.975)*sqrt(sd(SVM_NPV)/number.of.iterations)`]

4 b) iii) Using the two most important predictors

```{r,warning=FALSE, echo=TRUE, message=FALSE}
cancerdata_subset <- dplyr::select(cancerdata, Glucose, Resistin, Classification)
```

```{r, warning=FALSE, echo=TRUE, message=FALSE, cache=FALSE}
```

```
number.of.iterations=500

RF_AUC=matrix(NA, number.of.iterations, 1)

RF_acc=matrix(NA, number.of.iterations, 1)

RF_specificity=matrix(NA, number.of.iterations, 1)

RF_sensitivity=matrix(NA, number.of.iterations, 1)

RF_PPV=matrix(NA, number.of.iterations, 1)

RF_NPV=matrix(NA, number.of.iterations, 1)

#AUC, accuracy, specificity, sensitivity, PPV and NPV associated to applying RF


SVM_AUC=matrix(NA, number.of.iterations, 1)

SVM_acc=matrix(NA, number.of.iterations, 1)

SVM_specificity=matrix(NA, number.of.iterations, 1)

SVM_sensitivity=matrix(NA, number.of.iterations, 1)

SVM_PPV=matrix(NA, number.of.iterations, 1)

SVM_NPV=matrix(NA, number.of.iterations, 1)

#AUC, accuracy, specificity, sensitivity, PPV and NPV associated to applying SVM


LR_AUC=matrix(NA, number.of.iterations, 1)

LR_acc=matrix(NA, number.of.iterations, 1)

LR_specificity=matrix(NA, number.of.iterations, 1)

LR_sensitivity=matrix(NA, number.of.iterations, 1)

LR_PPV=matrix(NA, number.of.iterations, 1)

LR_NPV=matrix(NA, number.of.iterations, 1)

#AUC, accuracy, specificity, sensitivity, PPV and NPV associated to applying LR
```

```
for (iterationindex in 1:number.of.iterations){

set.seed(iterationindex) # controls the random process of splitting the data
splitteddata=splitter(cancerdata_subset, 0.7) # splits the data
trainset=splitteddata[[1]] # train set
testset=splitteddata[[2]] # test set

RF.prediction=RFclassifier(trainset, testset)
SVM.prediction=SVMclassifier(trainset, testset)
LR.prediction=LRclassifier(trainset, testset)

RF.evaluation=evaluateclassifier(RF.prediction, testset)
SVM.evaluation=evaluateclassifier(SVM.prediction, testset)
LR.evaluation=evaluateclassifier(LR.prediction, testset)


RF_AUC[iterationindex]=RF.evaluation[[1]]
RF_acc[iterationindex]=RF.evaluation[[2]]
RF_specificity[iterationindex]=RF.evaluation[[3]]
RF_sensitivity[iterationindex]=RF.evaluation[[4]]
RF_PPV[iterationindex]=RF.evaluation[[5]]
RF_NPV[iterationindex]=RF.evaluation[[6]]

SVM_AUC[iterationindex]=SVM.evaluation[[1]]
SVM_acc[iterationindex]=SVM.evaluation[[2]]
SVM_specificity[iterationindex]=SVM.evaluation[[3]]
SVM_sensitivity[iterationindex]=SVM.evaluation[[4]]
```

```
SVM_PPV[iterationindex]=SVM.evaluation[[5]]

SVM_NPV[iterationindex]=SVM.evaluation[[6]]


LR_AUC[iterationindex]=LR.evaluation[[1]]

LR_acc[iterationindex]=LR.evaluation[[2]]

LR_specificity[iterationindex]=LR.evaluation[[3]]

LR_sensitivity[iterationindex]=LR.evaluation[[4]]

LR_PPV[iterationindex]=LR.evaluation[[5]]

LR_NPV[iterationindex]=LR.evaluation[[6]]


}
```

So what we see that we obtain is

* for LR, the 95% confidence interval for the

    * AUC is [`r mean(LR_AUC)-qnorm(0.975)*sqrt(sd(LR_AUC)/number.of.iterations)`, `r mean(LR_AUC)+qnorm(0.975)*sqrt(sd(LR_AUC)/number.of.iterations)`]

    * accuracy is [`r mean(LR_acc)-qnorm(0.975)*sqrt(sd(LR_acc)/number.of.iterations)`, `r mean(LR_acc)+qnorm(0.975)*sqrt(sd(LR_acc)/number.of.iterations)`]

    * sensitivity is [`r mean(LR_sensitivity)-qnorm(0.975)*sqrt(sd(LR_sensitivity)/number.of.iterations)`, `r mean(LR_sensitivity)+qnorm(0.975)*sqrt(sd(LR_sensitivity)/number.of.iterations)`]

* specificity is [`r mean(LR_specificity)-qnorm(0.975)*sqrt(sd(LR_specificity)/number.of.iterations)`, `r mean(LR_specificity)+qnorm(0.975)*sqrt(sd(LR_specificity)/number.of.iterations)`]

* PPV is [`r mean(LR_PPV)-qnorm(0.975)*sqrt(sd(LR_PPV)/number.of.iterations)`, `r mean(LR_PPV)+qnorm(0.975)*sqrt(sd(LR_PPV)/number.of.iterations)`]

* NPV is [`r mean(LR_NPV)-qnorm(0.975)*sqrt(sd(LR_NPV)/number.of.iterations)`, `r mean(LR_NPV)+qnorm(0.975)*sqrt(sd(LR_NPV)/number.of.iterations)`]

* for RF, the 95% confidence interval for the

* AUC is [`r mean(RF_AUC)-qnorm(0.975)*sqrt(sd(RF_AUC)/number.of.iterations)`, `r mean(RF_AUC)+qnorm(0.975)*sqrt(sd(RF_AUC)/number.of.iterations)`]

* accuracy is [`r mean(RF_acc)-qnorm(0.975)*sqrt(sd(RF_acc)/number.of.iterations)`, `r mean(RF_acc)+qnorm(0.975)*sqrt(sd(RF_acc)/number.of.iterations)`]

* sensitivity is [`r mean(RF_sensitivity)-qnorm(0.975)*sqrt(sd(RF_sensitivity)/number.of.iterations)`, `r mean(RF_sensitivity)+qnorm(0.975)*sqrt(sd(RF_sensitivity)/number.of.iterations)`]

* specificity is [`r mean(RF_specificity)-qnorm(0.975)*sqrt(sd(RF_specificity)/number.of.iterations)`, `r mean(RF_specificity)+qnorm(0.975)*sqrt(sd(RF_specificity)/number.of.iterations)`]

* PPV is [`r mean(RF_PPV)-qnorm(0.975)*sqrt(sd(RF_PPV)/number.of.iterations)`, `r mean(RF_PPV)+qnorm(0.975)*sqrt(sd(RF_PPV)/number.of.iterations)`]

* NPV is [`r mean(RF_NPV)-qnorm(0.975)*sqrt(sd(RF_NPV)/number.of.iterations)`, `r mean(RF_NPV)+qnorm(0.975)*sqrt(sd(RF_NPV)/number.of.iterations)`]

* for SVM, the 95% confidence interval for the

    * AUC is [`r mean(SVM_AUC)-qnorm(0.975)*sqrt(sd(SVM_AUC)/number.of.iterations)`, `r mean(SVM_AUC)+qnorm(0.975)*sqrt(sd(SVM_AUC)/number.of.iterations)`]

    * accuracy is [`r mean(SVM_acc)-qnorm(0.975)*sqrt(sd(SVM_acc)/number.of.iterations)`, `r mean(SVM_acc)+qnorm(0.975)*sqrt(sd(SVM_acc)/number.of.iterations)`]

    * sensitivity is [`r mean(SVM_sensitivity)-qnorm(0.975)*sqrt(sd(SVM_sensitivity)/number.of.iterations)`, `r mean(SVM_sensitivity)+qnorm(0.975)*sqrt(sd(SVM_sensitivity)/number.of.iterations)`]

    * specificity is [`r mean(SVM_specificity)-qnorm(0.975)*sqrt(sd(SVM_specificity)/number.of.iterations)`, `r mean(SVM_specificity)+qnorm(0.975)*sqrt(sd(SVM_specificity)/number.of.iterations)`]

    * PPV is [`r mean(SVM_PPV)-qnorm(0.975)*sqrt(sd(SVM_PPV)/number.of.iterations)`, `r mean(SVM_PPV)+qnorm(0.975)*sqrt(sd(SVM_PPV)/number.of.iterations)`]

    * NPV is [`r mean(SVM_NPV)-qnorm(0.975)*sqrt(sd(SVM_NPV)/number.of.iterations)`, `r mean(SVM_NPV)+qnorm(0.975)*sqrt(sd(SVM_NPV)/number.of.iterations)`]

4 b) iv) Using the three most important predictors

````
```{r,warning=FALSE, echo=TRUE, message=FALSE}
cancerdata_subset <- dplyr::select(cancerdata, Glucose, Resistin, Age, Classification)
```
````

````
```{r, warning=FALSE, echo=TRUE, message=FALSE, cache=FALSE}
number.of.iterations=500
RF_AUC=matrix(NA, number.of.iterations, 1)
RF_acc=matrix(NA, number.of.iterations, 1)
RF_specificity=matrix(NA, number.of.iterations, 1)
RF_sensitivity=matrix(NA, number.of.iterations, 1)
RF_PPV=matrix(NA, number.of.iterations, 1)
RF_NPV=matrix(NA, number.of.iterations, 1)
#AUC, accuracy, specificity, sensitivity, PPV and NPV associated to applying RF
````
````

```
SVM_AUC=matrix(NA, number.of.iterations, 1)

SVM_acc=matrix(NA, number.of.iterations, 1)

SVM_specificity=matrix(NA, number.of.iterations, 1)

SVM_sensitivity=matrix(NA, number.of.iterations, 1)

SVM_PPV=matrix(NA, number.of.iterations, 1)

SVM_NPV=matrix(NA, number.of.iterations, 1)

#AUC, accuracy, specificity, sensitivity, PPV and NPV associated to applying SVM


LR_AUC=matrix(NA, number.of.iterations, 1)

LR_acc=matrix(NA, number.of.iterations, 1)

LR_specificity=matrix(NA, number.of.iterations, 1)

LR_sensitivity=matrix(NA, number.of.iterations, 1)

LR_PPV=matrix(NA, number.of.iterations, 1)

LR_NPV=matrix(NA, number.of.iterations, 1)

#AUC, accuracy, specificity, sensitivity, PPV and NPV associated to applying LR




for (iterationindex in 1:number.of.iterations){


set.seed(iterationindex) # controls the random process of splitting the data

splitteddata=splitter(cancerdata_subset, 0.7) # splits the data

trainset=splitteddata[[1]] # train set

testset=splitteddata[[2]] # test set


RF.prediction=RFclassifier(trainset, testset)
```

```
SVM.prediction=SVMclassifier(trainset, testset)
LR.prediction=LRclassifier(trainset, testset)

RF.evaluation=evaluateclassifier(RF.prediction, testset)
SVM.evaluation=evaluateclassifier(SVM.prediction, testset)
LR.evaluation=evaluateclassifier(LR.prediction, testset)


RF_AUC[iterationindex]=RF.evaluation[[1]]
RF_acc[iterationindex]=RF.evaluation[[2]]
RF_specificity[iterationindex]=RF.evaluation[[3]]
RF_sensitivity[iterationindex]=RF.evaluation[[4]]
RF_PPV[iterationindex]=RF.evaluation[[5]]
RF_NPV[iterationindex]=RF.evaluation[[6]]

SVM_AUC[iterationindex]=SVM.evaluation[[1]]
SVM_acc[iterationindex]=SVM.evaluation[[2]]
SVM_specificity[iterationindex]=SVM.evaluation[[3]]
SVM_sensitivity[iterationindex]=SVM.evaluation[[4]]
SVM_PPV[iterationindex]=SVM.evaluation[[5]]
SVM_NPV[iterationindex]=SVM.evaluation[[6]]

LR_AUC[iterationindex]=LR.evaluation[[1]]
LR_acc[iterationindex]=LR.evaluation[[2]]
LR_specificity[iterationindex]=LR.evaluation[[3]]
LR_sensitivity[iterationindex]=LR.evaluation[[4]]
LR_PPV[iterationindex]=LR.evaluation[[5]]
```

LR_NPV[iterationindex]=LR.evaluation[[6]]

}

```

So what we see that we obtain is

* for LR, the 95% confidence interval for the

    * AUC is [`r mean(LR_AUC)-qnorm(0.975)*sqrt(sd(LR_AUC)/number.of.iterations)`, `r mean(LR_AUC)+qnorm(0.975)*sqrt(sd(LR_AUC)/number.of.iterations)`]

    * accuracy is [`r mean(LR_acc)-qnorm(0.975)*sqrt(sd(LR_acc)/number.of.iterations)`, `r mean(LR_acc)+qnorm(0.975)*sqrt(sd(LR_acc)/number.of.iterations)`]

    * sensitivity is [`r mean(LR_sensitivity)-qnorm(0.975)*sqrt(sd(LR_sensitivity)/number.of.iterations)`, `r mean(LR_sensitivity)+qnorm(0.975)*sqrt(sd(LR_sensitivity)/number.of.iterations)`]

    * specificity is [`r mean(LR_specificity)-qnorm(0.975)*sqrt(sd(LR_specificity)/number.of.iterations)`, `r mean(LR_specificity)+qnorm(0.975)*sqrt(sd(LR_specificity)/number.of.iterations)`]

    * PPV is [`r mean(LR_PPV)-qnorm(0.975)*sqrt(sd(LR_PPV)/number.of.iterations)`, `r mean(LR_PPV)+qnorm(0.975)*sqrt(sd(LR_PPV)/number.of.iterations)`]

    * NPV is [`r mean(LR_NPV)-qnorm(0.975)*sqrt(sd(LR_NPV)/number.of.iterations)`, `r mean(LR_NPV)+qnorm(0.975)*sqrt(sd(LR_NPV)/number.of.iterations)`]
```

* for RF, the 95% confidence interval for the

    * AUC is [`r mean(RF_AUC)-qnorm(0.975)*sqrt(sd(RF_AUC)/number.of.iterations)`, `r mean(RF_AUC)+qnorm(0.975)*sqrt(sd(RF_AUC)/number.of.iterations)`]

    * accuracy is [`r mean(RF_acc)-qnorm(0.975)*sqrt(sd(RF_acc)/number.of.iterations)`, `r mean(RF_acc)+qnorm(0.975)*sqrt(sd(RF_acc)/number.of.iterations)`]

    * sensitivity is [`r mean(RF_sensitivity)-qnorm(0.975)*sqrt(sd(RF_sensitivity)/number.of.iterations)`, `r mean(RF_sensitivity)+qnorm(0.975)*sqrt(sd(RF_sensitivity)/number.of.iterations)`]

    * specificity is [`r mean(RF_specificity)-qnorm(0.975)*sqrt(sd(RF_specificity)/number.of.iterations)`, `r mean(RF_specificity)+qnorm(0.975)*sqrt(sd(RF_specificity)/number.of.iterations)`]

    * PPV is [`r mean(RF_PPV)-qnorm(0.975)*sqrt(sd(RF_PPV)/number.of.iterations)`, `r mean(RF_PPV)+qnorm(0.975)*sqrt(sd(RF_PPV)/number.of.iterations)`]

    * NPV is [`r mean(RF_NPV)-qnorm(0.975)*sqrt(sd(RF_NPV)/number.of.iterations)`, `r mean(RF_NPV)+qnorm(0.975)*sqrt(sd(RF_NPV)/number.of.iterations)`]

* for SVM, the 95% confidence interval for the

    * AUC is [`r mean(SVM_AUC)-qnorm(0.975)*sqrt(sd(SVM_AUC)/number.of.iterations)`, `r mean(SVM_AUC)+qnorm(0.975)*sqrt(sd(SVM_AUC)/number.of.iterations)`]

* accuracy is [`r mean(SVM_acc)-qnorm(0.975)*sqrt(sd(SVM_acc)/number.of.iterations)`, `r mean(SVM_acc)+qnorm(0.975)*sqrt(sd(SVM_acc)/number.of.iterations)`]

* sensitivity is [`r mean(SVM_sensitivity)-qnorm(0.975)*sqrt(sd(SVM_sensitivity)/number.of.iterations)`, `r mean(SVM_sensitivity)+qnorm(0.975)*sqrt(sd(SVM_sensitivity)/number.of.iterations)`]

* specificity is [`r mean(SVM_specificity)-qnorm(0.975)*sqrt(sd(SVM_specificity)/number.of.iterations)`, `r mean(SVM_specificity)+qnorm(0.975)*sqrt(sd(SVM_specificity)/number.of.iterations)`]

* PPV is [`r mean(SVM_PPV)-qnorm(0.975)*sqrt(sd(SVM_PPV)/number.of.iterations)`, `r mean(SVM_PPV)+qnorm(0.975)*sqrt(sd(SVM_PPV)/number.of.iterations)`]

* NPV is [`r mean(SVM_NPV)-qnorm(0.975)*sqrt(sd(SVM_NPV)/number.of.iterations)`, `r mean(SVM_NPV)+qnorm(0.975)*sqrt(sd(SVM_NPV)/number.of.iterations)`]

4 b) iv) Using the four most important predictors

```{r,warning=FALSE, echo=TRUE, message=FALSE}

cancerdata_subset <- dplyr::select(cancerdata, Glucose, BMI, Age, Resistin, Classification)

```

```{r, warning=FALSE, echo=TRUE, message=FALSE, cache=FALSE}

number.of.iterations=500

RF_AUC=matrix(NA, number.of.iterations, 1)

RF_acc=matrix(NA, number.of.iterations, 1)

RF_specificity=matrix(NA, number.of.iterations, 1)

RF_sensitivity=matrix(NA, number.of.iterations, 1)

RF_PPV=matrix(NA, number.of.iterations, 1)

RF_NPV=matrix(NA, number.of.iterations, 1)
```

```
#AUC, accuracy, specificity, sensitivity, PPV and NPV associated to applying RF


SVM_AUC=matrix(NA, number.of.iterations, 1)

SVM_acc=matrix(NA, number.of.iterations, 1)

SVM_specificity=matrix(NA, number.of.iterations, 1)

SVM_sensitivity=matrix(NA, number.of.iterations, 1)

SVM_PPV=matrix(NA, number.of.iterations, 1)

SVM_NPV=matrix(NA, number.of.iterations, 1)

#AUC, accuracy, specificity, sensitivity, PPV and NPV associated to applying SVM


LR_AUC=matrix(NA, number.of.iterations, 1)

LR_acc=matrix(NA, number.of.iterations, 1)

LR_specificity=matrix(NA, number.of.iterations, 1)

LR_sensitivity=matrix(NA, number.of.iterations, 1)

LR_PPV=matrix(NA, number.of.iterations, 1)

LR_NPV=matrix(NA, number.of.iterations, 1)

#AUC, accuracy, specificity, sensitivity, PPV and NPV associated to applying LR




for (iterationindex in 1:number.of.iterations){


set.seed(iterationindex) # controls the random process of splitting the data

splitteddata=splitter(cancerdata_subset, 0.7) # splits the data

trainset=splitteddata[[1]] # train set

testset=splitteddata[[2]] # test set
```

```
RF.prediction=RFclassifier(trainset, testset)

SVM.prediction=SVMclassifier(trainset, testset)

LR.prediction=LRclassifier(trainset, testset)


RF.evaluation=evaluateclassifier(RF.prediction, testset)

SVM.evaluation=evaluateclassifier(SVM.prediction, testset)

LR.evaluation=evaluateclassifier(LR.prediction, testset)



RF_AUC[iterationindex]=RF.evaluation[[1]]

RF_acc[iterationindex]=RF.evaluation[[2]]

RF_specificity[iterationindex]=RF.evaluation[[3]]

RF_sensitivity[iterationindex]=RF.evaluation[[4]]

RF_PPV[iterationindex]=RF.evaluation[[5]]

RF_NPV[iterationindex]=RF.evaluation[[6]]


SVM_AUC[iterationindex]=SVM.evaluation[[1]]

SVM_acc[iterationindex]=SVM.evaluation[[2]]

SVM_specificity[iterationindex]=SVM.evaluation[[3]]

SVM_sensitivity[iterationindex]=SVM.evaluation[[4]]

SVM_PPV[iterationindex]=SVM.evaluation[[5]]

SVM_NPV[iterationindex]=SVM.evaluation[[6]]


LR_AUC[iterationindex]=LR.evaluation[[1]]

LR_acc[iterationindex]=LR.evaluation[[2]]

LR_specificity[iterationindex]=LR.evaluation[[3]]

LR_sensitivity[iterationindex]=LR.evaluation[[4]]
```

LR_PPV[iterationindex]=LR.evaluation[[5]]

LR_NPV[iterationindex]=LR.evaluation[[6]]

}

```

So what we see that we obtain is

* for LR, the 95% confidence interval for the

    * AUC is [`r mean(LR_AUC)-qnorm(0.975)*sqrt(sd(LR_AUC)/number.of.iterations)`, `r mean(LR_AUC)+qnorm(0.975)*sqrt(sd(LR_AUC)/number.of.iterations)`]

    * accuracy is [`r mean(LR_acc)-qnorm(0.975)*sqrt(sd(LR_acc)/number.of.iterations)`, `r mean(LR_acc)+qnorm(0.975)*sqrt(sd(LR_acc)/number.of.iterations)`]

    * sensitivity is [`r mean(LR_sensitivity)-qnorm(0.975)*sqrt(sd(LR_sensitivity)/number.of.iterations)`, `r mean(LR_sensitivity)+qnorm(0.975)*sqrt(sd(LR_sensitivity)/number.of.iterations)`]

    * specificity is [`r mean(LR_specificity)-qnorm(0.975)*sqrt(sd(LR_specificity)/number.of.iterations)`, `r mean(LR_specificity)+qnorm(0.975)*sqrt(sd(LR_specificity)/number.of.iterations)`]

    * PPV is [`r mean(LR_PPV)-qnorm(0.975)*sqrt(sd(LR_PPV)/number.of.iterations)`, `r mean(LR_PPV)+qnorm(0.975)*sqrt(sd(LR_PPV)/number.of.iterations)`]

    * NPV is [`r mean(LR_NPV)-qnorm(0.975)*sqrt(sd(LR_NPV)/number.of.iterations)`, `r mean(LR_NPV)+qnorm(0.975)*sqrt(sd(LR_NPV)/number.of.iterations)`]
```

* for RF, the 95% confidence interval for the

    * AUC is [`r mean(RF_AUC)-qnorm(0.975)*sqrt(sd(RF_AUC)/number.of.iterations)`, `r mean(RF_AUC)+qnorm(0.975)*sqrt(sd(RF_AUC)/number.of.iterations)`]

    * accuracy is [`r mean(RF_acc)-qnorm(0.975)*sqrt(sd(RF_acc)/number.of.iterations)`, `r mean(RF_acc)+qnorm(0.975)*sqrt(sd(RF_acc)/number.of.iterations)`]

    * sensitivity is [`r mean(RF_sensitivity)-qnorm(0.975)*sqrt(sd(RF_sensitivity)/number.of.iterations)`, `r mean(RF_sensitivity)+qnorm(0.975)*sqrt(sd(RF_sensitivity)/number.of.iterations)`]

    * specificity is [`r mean(RF_specificity)-qnorm(0.975)*sqrt(sd(RF_specificity)/number.of.iterations)`, `r mean(RF_specificity)+qnorm(0.975)*sqrt(sd(RF_specificity)/number.of.iterations)`]

    * PPV is [`r mean(RF_PPV)-qnorm(0.975)*sqrt(sd(RF_PPV)/number.of.iterations)`, `r mean(RF_PPV)+qnorm(0.975)*sqrt(sd(RF_PPV)/number.of.iterations)`]

    * NPV is [`r mean(RF_NPV)-qnorm(0.975)*sqrt(sd(RF_NPV)/number.of.iterations)`, `r mean(RF_NPV)+qnorm(0.975)*sqrt(sd(RF_NPV)/number.of.iterations)`]

* for SVM, the 95% confidence interval for the

    * AUC is [`r mean(SVM_AUC)-qnorm(0.975)*sqrt(sd(SVM_AUC)/number.of.iterations)`, `r mean(SVM_AUC)+qnorm(0.975)*sqrt(sd(SVM_AUC)/number.of.iterations)`]

* accuracy is [`r mean(SVM_acc)-qnorm(0.975)*sqrt(sd(SVM_acc)/number.of.iterations)`, `r mean(SVM_acc)+qnorm(0.975)*sqrt(sd(SVM_acc)/number.of.iterations)`]

* sensitivity is [`r mean(SVM_sensitivity)-qnorm(0.975)*sqrt(sd(SVM_sensitivity)/number.of.iterations)`, `r mean(SVM_sensitivity)+qnorm(0.975)*sqrt(sd(SVM_sensitivity)/number.of.iterations)`]

* specificity is [`r mean(SVM_specificity)-qnorm(0.975)*sqrt(sd(SVM_specificity)/number.of.iterations)`, `r mean(SVM_specificity)+qnorm(0.975)*sqrt(sd(SVM_specificity)/number.of.iterations)`]

* PPV is [`r mean(SVM_PPV)-qnorm(0.975)*sqrt(sd(SVM_PPV)/number.of.iterations)`, `r mean(SVM_PPV)+qnorm(0.975)*sqrt(sd(SVM_PPV)/number.of.iterations)`]

* NPV is [`r mean(SVM_NPV)-qnorm(0.975)*sqrt(sd(SVM_NPV)/number.of.iterations)`, `r mean(SVM_NPV)+qnorm(0.975)*sqrt(sd(SVM_NPV)/number.of.iterations)`]

4 b) v) Using the five most important predictors

```{r,warning=FALSE, echo=TRUE, message=FALSE}
cancerdata_subset <- dplyr::select(cancerdata, Glucose, BMI, Age, Resistin, HOMA, Classification)
```

```{r, warning=FALSE, echo=TRUE, message=FALSE, cache=FALSE}
number.of.iterations=500
RF_AUC=matrix(NA, number.of.iterations, 1)
RF_acc=matrix(NA, number.of.iterations, 1)
RF_specificity=matrix(NA, number.of.iterations, 1)
RF_sensitivity=matrix(NA, number.of.iterations, 1)
RF_PPV=matrix(NA, number.of.iterations, 1)
RF_NPV=matrix(NA, number.of.iterations, 1)
#AUC, accuracy, specificity, sensitivity, PPV and NPV associated to applying RF

SVM_AUC=matrix(NA, number.of.iterations, 1)
SVM_acc=matrix(NA, number.of.iterations, 1)
SVM_specificity=matrix(NA, number.of.iterations, 1)
SVM_sensitivity=matrix(NA, number.of.iterations, 1)
SVM_PPV=matrix(NA, number.of.iterations, 1)
SVM_NPV=matrix(NA, number.of.iterations, 1)
#AUC, accuracy, specificity, sensitivity, PPV and NPV associated to applying SVM

LR_AUC=matrix(NA, number.of.iterations, 1)
LR_acc=matrix(NA, number.of.iterations, 1)
LR_specificity=matrix(NA, number.of.iterations, 1)
```

```
LR_sensitivity=matrix(NA, number.of.iterations, 1)
LR_PPV=matrix(NA, number.of.iterations, 1)
LR_NPV=matrix(NA, number.of.iterations, 1)
#AUC, accuracy, specificity, sensitivity, PPV and NPV associated to applying LR



for (iterationindex in 1:number.of.iterations){

set.seed(iterationindex) # controls the random process of splitting the data
splitteddata=splitter(cancerdata_subset, 0.7) # splits the data
trainset=splitteddata[[1]] # train set
testset=splitteddata[[2]] # test set

RF.prediction=RFclassifier(trainset, testset)
SVM.prediction=SVMclassifier(trainset, testset)
LR.prediction=LRclassifier(trainset, testset)

RF.evaluation=evaluateclassifier(RF.prediction, testset)
SVM.evaluation=evaluateclassifier(SVM.prediction, testset)
LR.evaluation=evaluateclassifier(LR.prediction, testset)


RF_AUC[iterationindex]=RF.evaluation[[1]]
RF_acc[iterationindex]=RF.evaluation[[2]]
RF_specificity[iterationindex]=RF.evaluation[[3]]
RF_sensitivity[iterationindex]=RF.evaluation[[4]]
```

RF_PPV[iterationindex]=RF.evaluation[[5]]

RF_NPV[iterationindex]=RF.evaluation[[6]]

SVM_AUC[iterationindex]=SVM.evaluation[[1]]

SVM_acc[iterationindex]=SVM.evaluation[[2]]

SVM_specificity[iterationindex]=SVM.evaluation[[3]]

SVM_sensitivity[iterationindex]=SVM.evaluation[[4]]

SVM_PPV[iterationindex]=SVM.evaluation[[5]]

SVM_NPV[iterationindex]=SVM.evaluation[[6]]

LR_AUC[iterationindex]=LR.evaluation[[1]]

LR_acc[iterationindex]=LR.evaluation[[2]]

LR_specificity[iterationindex]=LR.evaluation[[3]]

LR_sensitivity[iterationindex]=LR.evaluation[[4]]

LR_PPV[iterationindex]=LR.evaluation[[5]]

LR_NPV[iterationindex]=LR.evaluation[[6]]

}

```

So what we see that we obtain is

* for LR, the 95% confidence interval for the

    * AUC is [`r mean(LR_AUC)-qnorm(0.975)*sqrt(sd(LR_AUC)/number.of.iterations)`, `r mean(LR_AUC)+qnorm(0.975)*sqrt(sd(LR_AUC)/number.of.iterations)`]
```

* accuracy is [`r mean(LR_acc)-qnorm(0.975)*sqrt(sd(LR_acc)/number.of.iterations)`, `r mean(LR_acc)+qnorm(0.975)*sqrt(sd(LR_acc)/number.of.iterations)`]

* sensitivity is [`r mean(LR_sensitivity)-qnorm(0.975)*sqrt(sd(LR_sensitivity)/number.of.iterations)`, `r mean(LR_sensitivity)+qnorm(0.975)*sqrt(sd(LR_sensitivity)/number.of.iterations)`]

* specificity is [`r mean(LR_specificity)-qnorm(0.975)*sqrt(sd(LR_specificity)/number.of.iterations)`, `r mean(LR_specificity)+qnorm(0.975)*sqrt(sd(LR_specificity)/number.of.iterations)`]

* PPV is [`r mean(LR_PPV)-qnorm(0.975)*sqrt(sd(LR_PPV)/number.of.iterations)`, `r mean(LR_PPV)+qnorm(0.975)*sqrt(sd(LR_PPV)/number.of.iterations)`]

* NPV is [`r mean(LR_NPV)-qnorm(0.975)*sqrt(sd(LR_NPV)/number.of.iterations)`, `r mean(LR_NPV)+qnorm(0.975)*sqrt(sd(LR_NPV)/number.of.iterations)`]

* for RF, the 95% confidence interval for the

* AUC is [`r mean(RF_AUC)-qnorm(0.975)*sqrt(sd(RF_AUC)/number.of.iterations)`, `r mean(RF_AUC)+qnorm(0.975)*sqrt(sd(RF_AUC)/number.of.iterations)`]

* accuracy is [`r mean(RF_acc)-qnorm(0.975)*sqrt(sd(RF_acc)/number.of.iterations)`, `r mean(RF_acc)+qnorm(0.975)*sqrt(sd(RF_acc)/number.of.iterations)`]

* sensitivity is [`r mean(RF_sensitivity)-qnorm(0.975)*sqrt(sd(RF_sensitivity)/number.of.iterations)`, `r mean(RF_sensitivity)+qnorm(0.975)*sqrt(sd(RF_sensitivity)/number.of.iterations)`]

* specificity is [`r mean(RF_specificity)-qnorm(0.975)*sqrt(sd(RF_specificity)/number.of.iterations)`, `r mean(RF_specificity)+qnorm(0.975)*sqrt(sd(RF_specificity)/number.of.iterations)`]

* PPV is [`r mean(RF_PPV)-qnorm(0.975)*sqrt(sd(RF_PPV)/number.of.iterations)`, `r mean(RF_PPV)+qnorm(0.975)*sqrt(sd(RF_PPV)/number.of.iterations)`]

* NPV is [`r mean(RF_NPV)-qnorm(0.975)*sqrt(sd(RF_NPV)/number.of.iterations)`, `r mean(RF_NPV)+qnorm(0.975)*sqrt(sd(RF_NPV)/number.of.iterations)`]

* for SVM, the 95% confidence interval for the

* AUC is [`r mean(SVM_AUC)-qnorm(0.975)*sqrt(sd(SVM_AUC)/number.of.iterations)`, `r mean(SVM_AUC)+qnorm(0.975)*sqrt(sd(SVM_AUC)/number.of.iterations)`]

* accuracy is [`r mean(SVM_acc)-qnorm(0.975)*sqrt(sd(SVM_acc)/number.of.iterations)`, `r mean(SVM_acc)+qnorm(0.975)*sqrt(sd(SVM_acc)/number.of.iterations)`]

* sensitivity is [`r mean(SVM_sensitivity)-qnorm(0.975)*sqrt(sd(SVM_sensitivity)/number.of.iterations)`, `r mean(SVM_sensitivity)+qnorm(0.975)*sqrt(sd(SVM_sensitivity)/number.of.iterations)`]

* specificity is [`r mean(SVM_specificity)-qnorm(0.975)*sqrt(sd(SVM_specificity)/number.of.iterations)`, `r mean(SVM_specificity)+qnorm(0.975)*sqrt(sd(SVM_specificity)/number.of.iterations)`]

* PPV is [`r mean(SVM_PPV)-qnorm(0.975)*sqrt(sd(SVM_PPV)/number.of.iterations)`, `r mean(SVM_PPV)+qnorm(0.975)*sqrt(sd(SVM_PPV)/number.of.iterations)`]

   * NPV is [`r mean(SVM_NPV)-qnorm(0.975)*sqrt(sd(SVM_NPV)/number.of.iterations)`, `r mean(SVM_NPV)+qnorm(0.975)*sqrt(sd(SVM_NPV)/number.of.iterations)`]

4 b) v) Using the six most important predictors

```{r,warning=FALSE, echo=TRUE, message=FALSE}

cancerdata_subset <- dplyr::select(cancerdata, Glucose, BMI, Age, Resistin,

                                HOMA, Leptin, Classification)

```

```{r, warning=FALSE, echo=TRUE, message=FALSE, cache=FALSE}

number.of.iterations=500

RF_AUC=matrix(NA, number.of.iterations, 1)

RF_acc=matrix(NA, number.of.iterations, 1)
```

```
RF_specificity=matrix(NA, number.of.iterations, 1)

RF_sensitivity=matrix(NA, number.of.iterations, 1)

RF_PPV=matrix(NA, number.of.iterations, 1)

RF_NPV=matrix(NA, number.of.iterations, 1)

#AUC, accuracy, specificity, sensitivity, PPV and NPV associated to applying RF


SVM_AUC=matrix(NA, number.of.iterations, 1)

SVM_acc=matrix(NA, number.of.iterations, 1)

SVM_specificity=matrix(NA, number.of.iterations, 1)

SVM_sensitivity=matrix(NA, number.of.iterations, 1)

SVM_PPV=matrix(NA, number.of.iterations, 1)

SVM_NPV=matrix(NA, number.of.iterations, 1)

#AUC, accuracy, specificity, sensitivity, PPV and NPV associated to applying SVM


LR_AUC=matrix(NA, number.of.iterations, 1)

LR_acc=matrix(NA, number.of.iterations, 1)

LR_specificity=matrix(NA, number.of.iterations, 1)

LR_sensitivity=matrix(NA, number.of.iterations, 1)

LR_PPV=matrix(NA, number.of.iterations, 1)

LR_NPV=matrix(NA, number.of.iterations, 1)

#AUC, accuracy, specificity, sensitivity, PPV and NPV associated to applying LR




for (iterationindex in 1:number.of.iterations){


set.seed(iterationindex) # controls the random process of splitting the data
```

```
splitteddata=splitter(cancerdata_subset, 0.7) # splits the data
trainset=splitteddata[[1]] # train set
testset=splitteddata[[2]] # test set

RF.prediction=RFclassifier(trainset, testset)
SVM.prediction=SVMclassifier(trainset, testset)
LR.prediction=LRclassifier(trainset, testset)

RF.evaluation=evaluateclassifier(RF.prediction, testset)
SVM.evaluation=evaluateclassifier(SVM.prediction, testset)
LR.evaluation=evaluateclassifier(LR.prediction, testset)


RF_AUC[iterationindex]=RF.evaluation[[1]]
RF_acc[iterationindex]=RF.evaluation[[2]]
RF_specificity[iterationindex]=RF.evaluation[[3]]
RF_sensitivity[iterationindex]=RF.evaluation[[4]]
RF_PPV[iterationindex]=RF.evaluation[[5]]
RF_NPV[iterationindex]=RF.evaluation[[6]]

SVM_AUC[iterationindex]=SVM.evaluation[[1]]
SVM_acc[iterationindex]=SVM.evaluation[[2]]
SVM_specificity[iterationindex]=SVM.evaluation[[3]]
SVM_sensitivity[iterationindex]=SVM.evaluation[[4]]
SVM_PPV[iterationindex]=SVM.evaluation[[5]]
SVM_NPV[iterationindex]=SVM.evaluation[[6]]
```

LR_AUC[iterationindex]=LR.evaluation[[1]]

LR_acc[iterationindex]=LR.evaluation[[2]]

LR_specificity[iterationindex]=LR.evaluation[[3]]

LR_sensitivity[iterationindex]=LR.evaluation[[4]]

LR_PPV[iterationindex]=LR.evaluation[[5]]

LR_NPV[iterationindex]=LR.evaluation[[6]]

}

```

So what we see that we obtain is

* for LR, the 95% confidence interval for the

    * AUC is [`r mean(LR_AUC)-qnorm(0.975)*sqrt(sd(LR_AUC)/number.of.iterations)`, `r mean(LR_AUC)+qnorm(0.975)*sqrt(sd(LR_AUC)/number.of.iterations)`]

    * accuracy is [`r mean(LR_acc)-qnorm(0.975)*sqrt(sd(LR_acc)/number.of.iterations)`, `r mean(LR_acc)+qnorm(0.975)*sqrt(sd(LR_acc)/number.of.iterations)`]

    * sensitivity is [`r mean(LR_sensitivity)-qnorm(0.975)*sqrt(sd(LR_sensitivity)/number.of.iterations)`, `r mean(LR_sensitivity)+qnorm(0.975)*sqrt(sd(LR_sensitivity)/number.of.iterations)`]

    * specificity is [`r mean(LR_specificity)-qnorm(0.975)*sqrt(sd(LR_specificity)/number.of.iterations)`, `r mean(LR_specificity)+qnorm(0.975)*sqrt(sd(LR_specificity)/number.of.iterations)`]
```

* PPV is [`r mean(LR_PPV)-qnorm(0.975)*sqrt(sd(LR_PPV)/number.of.iterations)`, `r mean(LR_PPV)+qnorm(0.975)*sqrt(sd(LR_PPV)/number.of.iterations)`]

* NPV is [`r mean(LR_NPV)-qnorm(0.975)*sqrt(sd(LR_NPV)/number.of.iterations)`, `r mean(LR_NPV)+qnorm(0.975)*sqrt(sd(LR_NPV)/number.of.iterations)`]

* for RF, the 95% confidence interval for the

  * AUC is [`r mean(RF_AUC)-qnorm(0.975)*sqrt(sd(RF_AUC)/number.of.iterations)`, `r mean(RF_AUC)+qnorm(0.975)*sqrt(sd(RF_AUC)/number.of.iterations)`]

  * accuracy is [`r mean(RF_acc)-qnorm(0.975)*sqrt(sd(RF_acc)/number.of.iterations)`, `r mean(RF_acc)+qnorm(0.975)*sqrt(sd(RF_acc)/number.of.iterations)`]

  * sensitivity is [`r mean(RF_sensitivity)-qnorm(0.975)*sqrt(sd(RF_sensitivity)/number.of.iterations)`, `r mean(RF_sensitivity)+qnorm(0.975)*sqrt(sd(RF_sensitivity)/number.of.iterations)`]

  * specificity is [`r mean(RF_specificity)-qnorm(0.975)*sqrt(sd(RF_specificity)/number.of.iterations)`, `r mean(RF_specificity)+qnorm(0.975)*sqrt(sd(RF_specificity)/number.of.iterations)`]

  * PPV is [`r mean(RF_PPV)-qnorm(0.975)*sqrt(sd(RF_PPV)/number.of.iterations)`, `r mean(RF_PPV)+qnorm(0.975)*sqrt(sd(RF_PPV)/number.of.iterations)`]

  * NPV is [`r mean(RF_NPV)-qnorm(0.975)*sqrt(sd(RF_NPV)/number.of.iterations)`, `r mean(RF_NPV)+qnorm(0.975)*sqrt(sd(RF_NPV)/number.of.iterations)`]

* for SVM, the 95% confidence interval for the

    * AUC is [`r mean(SVM_AUC)-qnorm(0.975)*sqrt(sd(SVM_AUC)/number.of.iterations)`, `r mean(SVM_AUC)+qnorm(0.975)*sqrt(sd(SVM_AUC)/number.of.iterations)`]

    * accuracy is [`r mean(SVM_acc)-qnorm(0.975)*sqrt(sd(SVM_acc)/number.of.iterations)`, `r mean(SVM_acc)+qnorm(0.975)*sqrt(sd(SVM_acc)/number.of.iterations)`]

    * sensitivity is [`r mean(SVM_sensitivity)-qnorm(0.975)*sqrt(sd(SVM_sensitivity)/number.of.iterations)`, `r mean(SVM_sensitivity)+qnorm(0.975)*sqrt(sd(SVM_sensitivity)/number.of.iterations)`]

    * specificity is [`r mean(SVM_specificity)-qnorm(0.975)*sqrt(sd(SVM_specificity)/number.of.iterations)`, `r mean(SVM_specificity)+qnorm(0.975)*sqrt(sd(SVM_specificity)/number.of.iterations)`]

    * PPV is [`r mean(SVM_PPV)-qnorm(0.975)*sqrt(sd(SVM_PPV)/number.of.iterations)`, `r mean(SVM_PPV)+qnorm(0.975)*sqrt(sd(SVM_PPV)/number.of.iterations)`]

    * NPV is [`r mean(SVM_NPV)-qnorm(0.975)*sqrt(sd(SVM_NPV)/number.of.iterations)`, `r mean(SVM_NPV)+qnorm(0.975)*sqrt(sd(SVM_NPV)/number.of.iterations)`]

4 c) Final logistic model

````
```{r,warning=FALSE, echo=TRUE, message=FALSE}

cancerdata_subset <- dplyr::select(cancerdata, Glucose, BMI, Age, Resistin, Classification)

```
````

Let's build a logistic regression model for the 4 most important predictors:

```{r, warning=FALSE, echo=TRUE, message=FALSE}

levels(cancerdata_subset)=c(1, 2)

glmmodel=glm(Classification~.,data=cancerdata_subset,family='binomial')

summary(glmmodel)

```

What we have here are the beta-coefficients (means and standard errors, the z-values, the p-values) and the odds ratios+confidence intervals:

```{r, warning=FALSE, echo=FALSE, message=FALSE}

glmtable=cbind(coef(summary(glmmodel)), exp(coef(summary(glmmodel))[, 1]), exp(confint(glmmodel)))

colnames(glmtable)[5]="Odds ratios"

round(glmtable, digits=3)

```

As for McFaden's pseudo R square and the result of Hosmer-Lemeshow goodness of fit test:

```{r, warning=FALSE, echo=FALSE, message=FALSE}

pR2(glmmodel)[4]

hoslem.test(as.numeric(cancerdata_subset$Classification)-1, fitted(glmmodel), g=10)


```

5. Appendix

The auxiliary functions built for this analysis are:

````
```{r, warning=FALSE, echo=TRUE, message=FALSE}
splitter
LRclassifier
SVMclassifier
RFclassifier
evaluateclassifier
```
````

Auxiliary Functions

```
splitter <- function(datatosplit, trainsizeinpercentage) {




  cancerdatatosplit=datatosplit[datatosplit$Classification==2,]
  controlsdatatosplit=datatosplit[datatosplit$Classification==1,]

  n=nrow(cancerdatatosplit)
  ntrain = round(n*trainsizeinpercentage) # size of train set
  tindex = sample(n,ntrain)          # indices of training samples

  cancertrainset = cancerdatatosplit[tindex,]
  cancertestset = cancerdatatosplit[-tindex,]

  n=nrow(controlsdatatosplit)
  ntrain = round(n*trainsizeinpercentage) # size of train set
  tindex = sample(n,ntrain)          # indices of training samples

  controlstrainset = controlsdatatosplit[tindex,]
  controlstestset = controlsdatatosplit[-tindex,]


  trainset=rbind(cancertrainset, controlstrainset)
  testset=rbind(controlstestset, cancertestset)
```

```
  return(list(trainset, testset))
}

evaluateclassifier <- function(prediction, testset) {

  real.diagnosis=testset$Classification

  roccurve=roc(real.diagnosis, prediction) # ROC curve
  AUCclassification=auc(roccurve)
  lowerbound_ROC=ci(roccurve)[1]
  upperbound_ROC=ci(roccurve)[3]

  threshold=roccurve$threshold[which.max(roccurve$sensitivities+roccurve$specificities)] #
threshold which optimises Youden's index
  binary.classification=as.numeric(prediction>threshold)+1 # binary classification (optimising
Youden's index)

  classtable=table(real.diagnosis, binary.classification)
  d=classtable[1, 1]
  c=classtable[1, 2]
  b=classtable[2, 1]
  a=classtable[2, 2]

  accuracy=(a+d)/sum(classtable) # accuracy of classification
  specificity=roccurve$specificities[which.max(roccurve$sensitivities+roccurve$specificities)]
```

```
  sensitivity=roccurve$sensitivities[which.max(roccurve$sensitivities+roccurve$specificities)]

  PPV=a/(a+c)

  NPV=d/(d+b)


  return(list(AUCclassification, accuracy, specificity, sensitivity, PPV, NPV, lowerbound_ROC,
upperbound_ROC))

}


LRclassifier <- function(trainset, testset) {


  LRfit = glm(Classification~.,data=trainset,family='binomial') # build model with LR


  cancer.probability = predict.glm(LRfit,testset[c(-ncol(testset))], type = 'response') # predict cancer
probabilities for test set


  return(cancer.probability)

}
```

```
RFclassifier <- function(trainset, testset) {

  RFfit = randomForest(Classification ~ .,   data=trainset)

  cancer.probability = predict(RFfit,testset[c(-ncol(testset))],  type = "vote")

  cancer.probability=cancer.probability[, 2]

  return(cancer.probability)
}

SVMclassifier <- function(trainset, testset) {

  SVMfit=svm(Classification~.,data=trainset, probability = TRUE)

  cancer.probability = predict(SVMfit,testset[c(-ncol(testset))], probability=TRUE)

  cancer.probability=attr(cancer.probability, "prob")[,1]
```

```
  return(cancer.probability)
}
```
